# Ovarian Matrisome Dynamics and αvβ3‐Mediated Regulation in Early Follicular Development

**DOI:** 10.1002/advs.202507314

**Published:** 2026-03-14

**Authors:** Tong Wu, Yanzhi Feng, Qingqing Zhu, Ting Jiang, Yueyue Gao, Kebing Nie, Xianan Tang, Ying Chen, Dianxing Hu, Xudan Yang, Shixuan Wang, Meng Wu, Jinjin Zhang

**Affiliations:** ^1^ National Clinical Research Center For Obstetrical and Gynecological Diseases Tongji Hospital Huazhong University of Science and Technology Wuhan P. R. China; ^2^ CAS Key Laboratory of Urban Pollutant Conversion Department of Environmental Science and Technology University of Science and Technology of China Hefei P. R. China

**Keywords:** early follicles development, extracellular matrix, folliculogenesis, matrisome, ovary

## Abstract

Early follicular development (EFD) spans the growth from primordial to small antral stages and is crucial for maintaining ovarian function. However, the specific role of matrisome during this process remains largely undefined. We perform integrated transcriptomic and proteomic analyses of prepubertal mouse ovaries to delineate the dynamics of the matrisome during EFD. Cross‐species comparison reveals a significant enrichment of matrisome‐associated items in mice, sheep, and pigs. We employ instrumental nanoindentation and Raman spectroscopy to obtain mechanical, topographical, and biochemical spectral data. And integrin αvβ3 is identified as a critical receptor mediating matrisome signals to follicles. Functional inhibition of αvβ3 exhibits a dual effect: it promotes primordial follicle activation, yet concurrently induces follicular atresia. Moreover, disruption of matrisome signals hinders secondary follicles growth by attenuating the cytoskeletal integrity and viability of granulosa cells; it also compromises oocyte maturation by disturbing the meiosis progression and mitochondrial function. Importantly, the role of matrisome is underscored in human ovarian tissues, which exhibited similar collagen remodeling and stage‐specific αvβ3 expression. Collectively, our work constructs a multi‐dimensional atlas of matrisome during EFD and determines αvβ3 as a pivotal mediator of matrisome‐follicle crosstalk, offering mechanistic insights into ovarian dysfunction and impaired follicular development.

## Graphical abstract

The matrisome undergoes dynamic remodeling during early follicular development. Integrin αvβ3 mediates matrisome signals, regulating primordial follicle activation/atresia and secondary follicle growth via Hippo/mTOR pathways, with conserved roles in human ovaries, offering therapeutic targets for ovarian disorders.

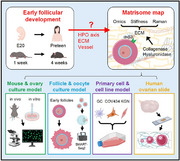

## 1 Introduction

Female fertility and health are fundamentally dependent on ovarian function, which in turn is governed by the precise orchestration of early follicular development (EFD). EFD encompasses the coordinated growth of follicles from the primordial through the primary, secondary and small antral stages [1], a process critical for puberty, ovulation and menstrual regulation. Aberrant EFD has been implicated in various ovarian disorders, including polycystic ovary syndrome, premature ovarian insufficiency, and diminished ovarian reserve [1, 2, 3]. Despite its clinical importance, the factors regulating EFD, particularly those within the tissue microenvironment, remain poorly understood.

Matrisome refers to the ensemble of more than 1,000 extracellular matrix (ECM) proteins, and is pivotal in tissue structure and function. Recent advancements in matrisome research have led to the establishment of comprehensive databases and atlases, providing insights into its biochemical and mechanical properties in both physiological and pathological contexts [4, 5, 6]. In terms of ovarian research, Ouni et al. pioneered the mapping of the ovarian matrisome in peri‐menopausal women in 2019, laying the groundwork for ovarian matrisome [7]. Their subsequent studies further showed the ECM remodeling of human ovaries from prepuberty until menopause, creating the first ovarian proteomic codex [8]. Proteomic analysis on decellularized porcine and bovine ovaries provided comprehensive matrisome proteins for constructing artificial ovaries [9, 10]. While these foundational studies have cataloged matrisome components, they remain largely descriptive. A critical knowledge gap remains: the dynamic changes of the matrisome during EFD and its regulatory mechanisms in determining follicular fate. Furthermore, prevailing omics technologies, while identifying compositional changes, fail to capture the critical biomechanical and biophysical properties of the ECM, which are essential for fully understanding cell‐ECM crosstalk and informing tissue engineering strategies.

Building on the preliminary characterization of the ovarian matrisome composition by existing studies, two critical gaps remain: the dynamic change patterns of the matrisome during EFD and its regulatory mechanisms. To bridge these gaps, we constructed a multi‐dimensional map of the matrisome across EFD by integrating atomic force microscopy, Raman spectroscopy, and transcriptomic sequencing. This approach uniquely captures its evolving mechanical, compositional, and spatial properties. We further identified integrin αvβ3 as a key receptor mediating matrisome‐follicles interactions and revealed conserved matrisome dynamics in both sheep and human ovarian. Our findings not only advance the fundamental understanding of ovarian biology by linking matrisome dynamics to follicular fate but also identify integrin αvβ3 as a potential therapeutic target. This holds promise for treating ovarian disorders characterized by impaired EFD, such as PCOS and POI.

## 2 Results

### 2.1 Matrisome Compositional Dynamics and Cross‐species Conservation During Early Follicular Development

To delineate the matrisome's role in EFD, we first established a mouse model that captures a complete follicular wave while minimizing confounding effects from ovulation. Histological and molecular characterization of ovaries from 3‐day‐ to 5‐week‐old mice identified the 1‐ and 4‐week stages as optimal for EFD studies (Figure S1)

**FIGURE 1:**
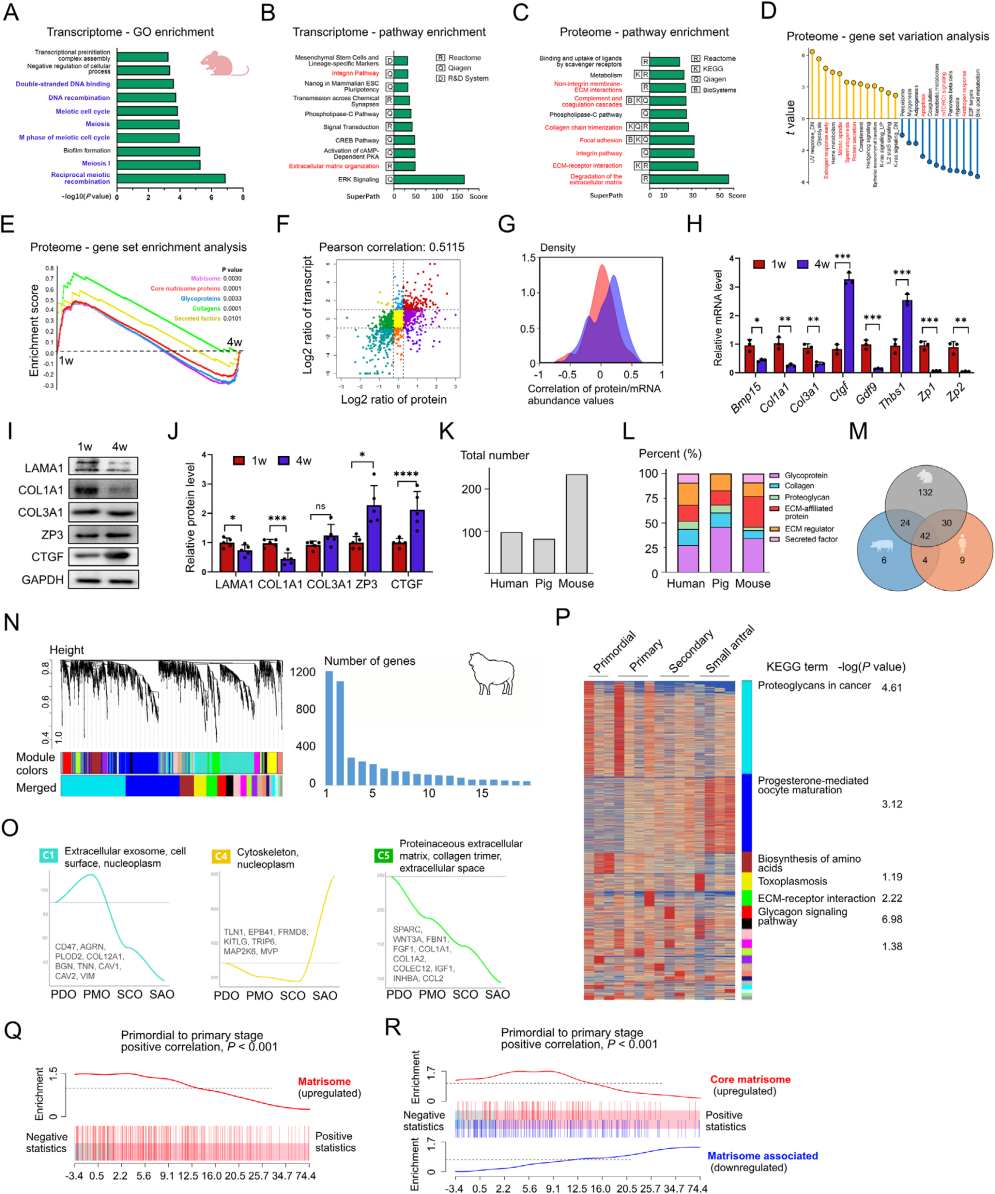
Dynamic Matrisome Changes Correlate With Early Follicular Development (EFD): (A) Gene ontology (GO)‐biological process enrichment analysis of differentially expressed gene (DEGs) in ovaries from 1‐week‐old and 4‐week‐old C57BL/6J mice. Follicular development‐associated items were marked blue. (B–C) Pathway enrichment analysis of DEGs and differentially expressed protein (DEPs) using databases including Reactome, KEGG, Qiagen, R&D System, and BioSystems. Matrisome‐related pathways were marked red. (D) Gene set variation analysis and (E) gene set enrichment analysis of DEPs in ovaries from 1‐week‐old and 4‐week‐old C57BL/6J mice. Matrisome‐related pathways were marked red. (F) Nine‐quadrant plot depicting the correlation between protein and transcript levels. The Pearson correlation coefficient is 0.5115, and the *p* value is 0. (G) Density plot illustrating the distribution of correlation values between protein and mRNA abundance. The overlapping and non‐overlapping regions of the two density curves depict the similarity and differences in the correlation patterns of protein and mRNA abundance, respectively. (H–J) Validation of key DEGs (*n* = 3) and DEPs (*n* = 6) via real‐time quantitative polymerase chain reaction and western blotting, respectively. (K–L) Total number and percentage of matrisome proteins in human, pig, and mouse ovaries. Human and pig matrisome data were sourced from published studies. Matrisome proteins were categorized into six groups: core matrisome and matrisome‐associated proteins based on MatrisomeDB 2.0. (M) Venn diagram showing overlapping and species‐specific matrisome proteins among human, pig, and mouse ovaries. (N) Weighted gene co‐expression network analysis cluster tree of DEGs in sheep oocytes, identifying 19 co‐expressed gene modules. (O) Cell component enrichment analysis of the abundant modules, and averaged expression levels of representative 30 genes were shown. (P) Heatmap showing gene expression of 19 modules of oocyte samples across four developmental points. Color intensity indicates normalized gene expression (z‐score). Right panel: KEGG pathway enrichment analysis of key modules. (Q–R) ROAST enrichment plot showed a positive correlation between matrisome gene sets and primordial‐primary transition. Data are presented as mean ± standard deviation (SD). Statistical analysis was performed using Student's *t* test (H, J). All tests were two tailed. *, *p* < 0.05; **, *p* < 0.01; ***, *p* < 0.001; ****, *p* < 0.0001.

To explore potential associations between matrisome and EFD, we performed transcriptomic and proteomic profiling of ovaries from 1‐week‐old and 4‐week‐old mice [11]. Functional annotation analyses pointed to matrisome‐associated biological processes, including integrin signaling, ECM organization, ECM degradation, ECM‐receptor interactions, and collagen chain trimerization (Figure 1A–D, Figure S2), which implies that matrisome may play a regulatory role in EFD. Matrisome‐related gene sets showed differential expression during EFD (Figure 1E), and several matrisome genes (e.g., *Gm5483*, *Stfa1*, *Stfa3*, *S100a8*, *Thbs4*) exhibited correlated expression patterns in both omics’ datasets (Figure 1F–G). The reliability of the high‐throughput sequencing data was supported by consistent expression trends of key differentially expressed mRNAs and proteins (Figure 1H–J). For mRNA levels, *Col1* and *Col3* showed significant down‐regulation at 4 weeks compared to 1 week, with *Ctgf* exhibiting the most dramatic increase. Meanwhile, most protein levels of corresponding components match their mRNA expression patterns

To extend these findings beyond mice, we analyzed ovarian matrisome composition across humans, pigs, and mice. Notably, we conducted proteomic profiling on intact ovaries from 1‐week‐old and 4‐week‐old C57BL/6J mice and found that murine ovaries contained more matrisome proteins than human and porcine ovaries (Figure 1K, Table S3), with glycoproteins (34%) and ECM regulators (30%) as the most abundant categories (Figure 1L, Figure S3). Cross‐species comparison identified 50 overlapping ECM components, including A2M, FN1, COL1A2, COL4A2, TGFBI, etc. (Figure 1M, Table S1), complementing our murine omics description of EFD‐associated matrisome.

To verify evolutionary conservation of matrisome function in EFD, we analyzed matrisome dynamics during sheep follicle development (primordial to antral stages) using WGCNA on 15 follicular transcriptomes (Figure 1N). Nineteen stage‐specific co‐expression modules were identified, with three modules showing relevance to matrisome function. Module C1 was enriched for extracellular functions (extracellular exosomes, cell surface; Figure 1O), with proteoglycans in cancer as the top statistically significant pathway (Figure 1P). Module C4 contained cytoskeleton‐related genes, while Module C5 focused on proteinaceous ECM, collagen trimerization, and extracellular space (Figure 1O), and enriching for ECM‐receptor interaction pathways (focal adhesion, protein digestion and absorption; Figure 1P). To further clarify matrisome's role in primordial follicle activation, unbiased ROAST analysis showed oocyte DEG expression profiles strongly correlated with matrisome and core matrisome signatures (Figure 1Q–R). Collectively, these multi‐species data demonstrate that dynamic matrisome remodeling is a conserved hallmark of EFD, providing a compositional framework for its functional investigation.

### 2.2 Structural Remodeling and Functional Validation of the Matrisome in Driving Follicle Activation

**FIGURE 2:**
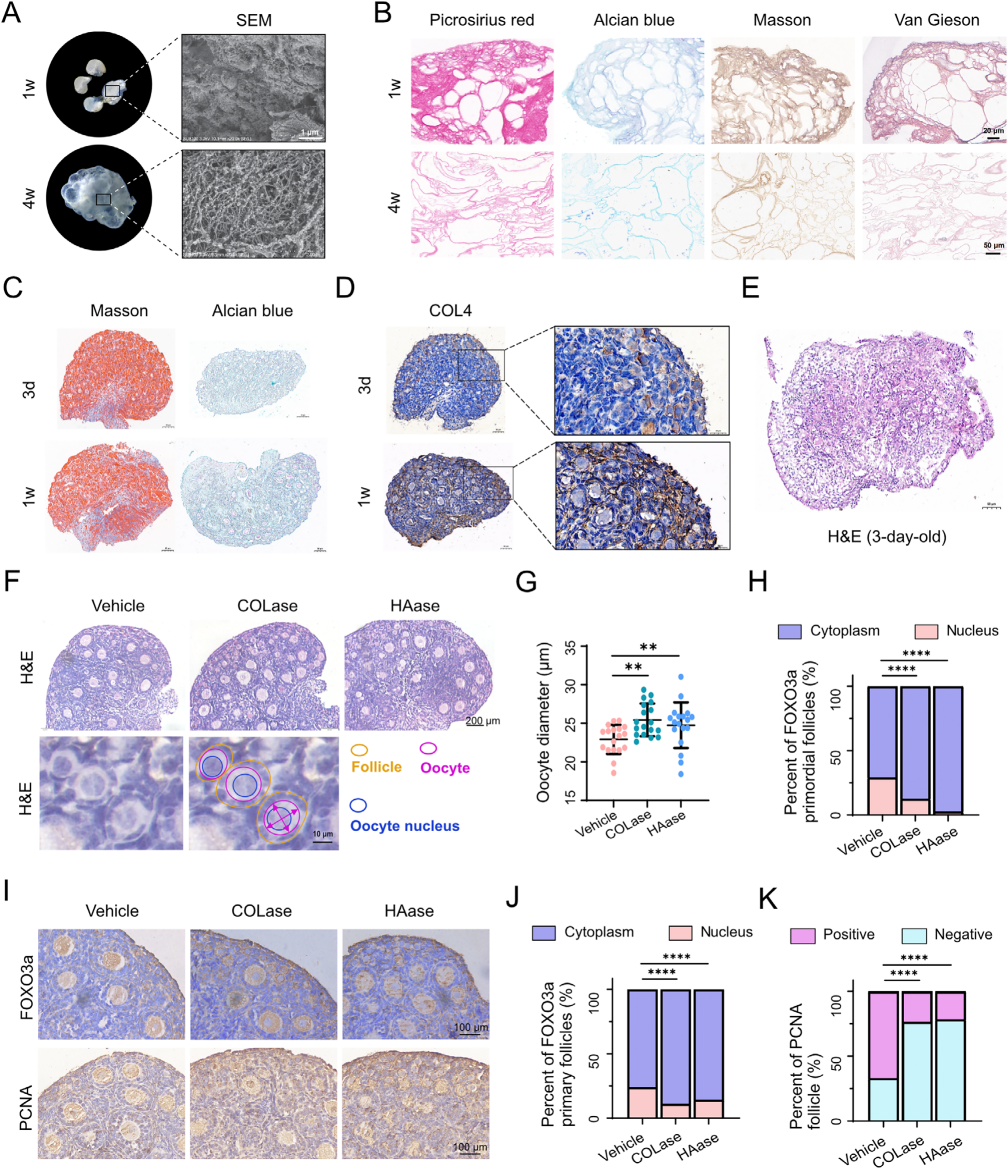
Disruption of Matrisome Proteins Accelerates EFD (A) Gross morphology and scanning electron microscopy (SEM) images of decellularized ovaries from 1‐week‐old and 4‐week‐old mice. (B) Connective tissue staining of decellularized ovarian extracellular matrix: Sirius red for collagen fibers, Alcian blue for acidic glycosaminoglycans, Masson's trichrome for collagen/elastin, and Van Gieson staining for collagen. (C) Masson's trichrome and Alcian blue staining of 3‐day‐old and 1‐week‐old mouse ovarian tissues. (D) Immunohistochemical staining for collagen IV in 3d and 1w mouse ovaries. (E) H&E staining of 3‐day‐old mouse ovarian tissues following exposure to low‐concentration collagenase, demonstrating overt tissue fragmentation and loss of follicle structural integrity. (F) H&E staining of ovarian tissue treated with vehicle, collagenase (COLase), or hyaluronidase (HAase), with lower schematic diagram illustrating the measurement of oocyte diameter and follicular components in primordial follicles. (G) Oocyte diameter in primordial follicles from neonatal ovaries treated with collagenase (*n* = 18). (H–J) Immunohistochemical staining and analysis of FOXO3A in oocyte cytoplasm and nucleus after ECM proteolysis. (K) Immunohistochemical staining of PCNA (proliferating cell nuclear antigen) in negative and positive ovarian tissue, with quantification of PCNA‐positive follicles. Data are presented as mean ±SD. Statistical analysis was performed using one‐way ANOVA (G) or Pearson's chi‐squared test (H, J, K). All tests were two tailed. **, *p* < 0.01; ****, *p* < 0.0001.

To further decipher the biophysical mechanisms underlying matrisome function during EFD, we conducted high‐resolution nanomechanical mapping and structural analysis of 1‐ to 5‐week‐old ovaries. Atomic force microscopy (AFM) revealed relatively stable ovarian stiffness throughout EFD (Figure 2A–C), consistent with previous reports [12]. However, nanoscale surface topography analysis identified increased roughness at 3 weeks of age (Figure 2D–E), suggesting volume expansion of early follicles and active ECM remodeling within the ovaries. Complementary Raman spectroscopy with non‐negative matrix factorization enabled in‐situ detection of matrisome signals at nanometer resolution (Figure 2F). The uniform and weak distribution of matrisome observed in 1‐week‐old ovaries suggested that ECM remodeling is inactive (Figure 2G). In contrast, 4‐week‐old ovaries exhibited distinct collagen and GAG signals outside ovarian cells (Figure 2H), indicating ECM maturation and enhanced cell‐matrix interactions.

**FIGURE 3:**
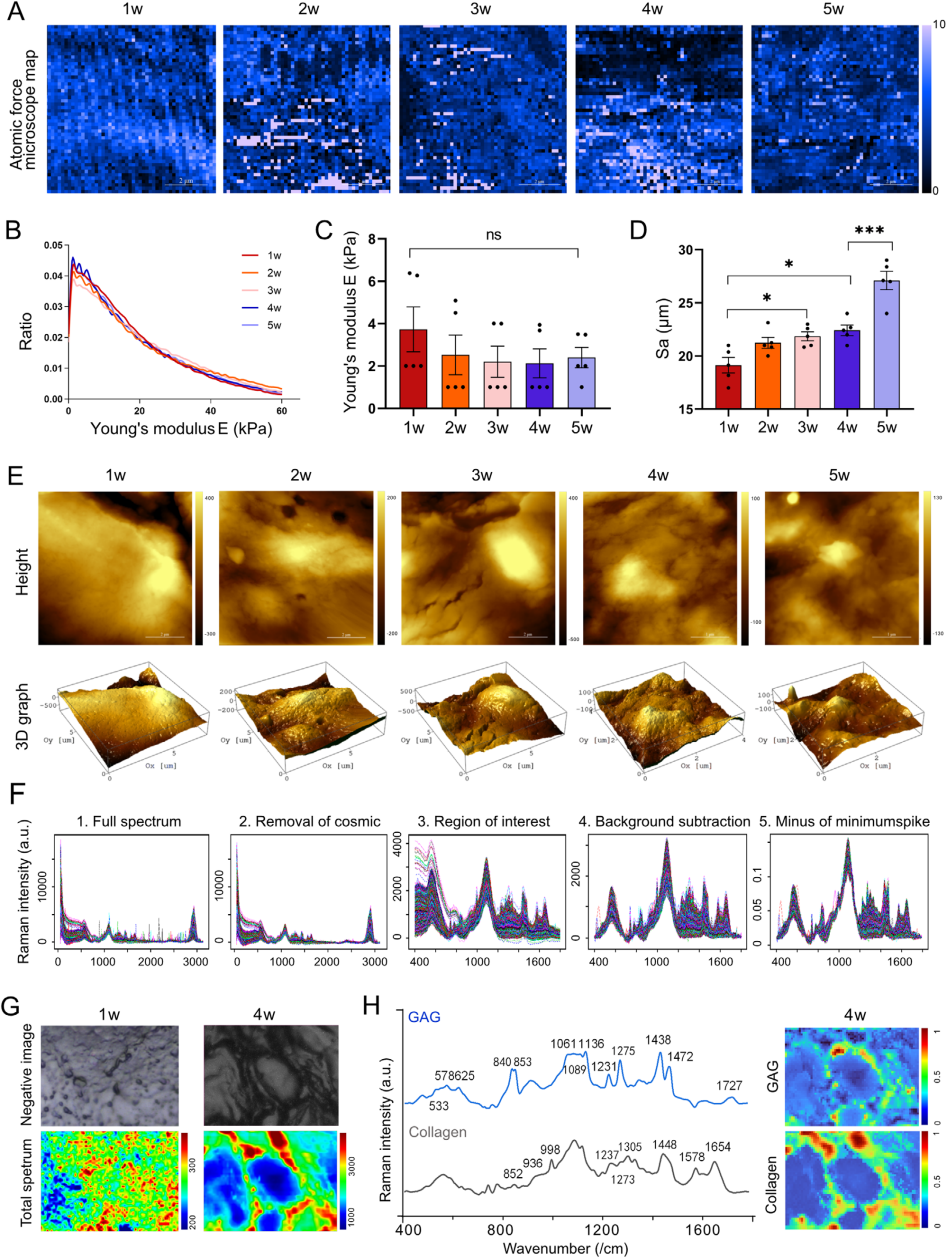
Physical Characterization Landscape of Matrisome Map During EFD: (A) Representative atomic force microscopy (AFM) maps of ovaries from 1‐ to 5‐week‐old C57BL/6J mice. (B) Statistical distribution of ovarian stiffness (*n* = 5). (C) Quantification of Young's modulus (kPa) across 1‐ to 5‐week‐old ovaries (*n* = 5). (D) Surface roughness calculated from local height variations of the high‐resolution maps. (E) AFM height profiles and 3D reconstructions of ovarian surfaces, illustrating changes in surface topography during EFD. (F) Workflow for non‐negative matrix factorization analysis of Raman spectra. (G) Raman mappings of 1‐week‐old and 4‐week‐old ovaries to visualize the distribution of extracellular matrix components. (H) Component spectra of glycosaminoglycan and collagen derived from non‐negative matrix factorization, with characteristic peaks labeled. Data are presented as mean ± SD. Statistical analysis was performed using one‐way (C, D). All tests were two tailed. *, *p* < 0.05; ***, *p* < 0.001; ns, not significant.

Having established compositional and structural associations, we next asked whether such remodeling is functionally instructive for follicle activation. Decellularization of ovarian tissues revealed a marked reduction in cortical collagen fibers, acidic glycosaminoglycans (GAGs), and elastin fibers density from 1 and 4 weeks of age (Figure 3A–B), suggesting that physical ECM loosening accompanies follicular development.

To test this functionally, we performed controlled ECM proteolysis experiments using collagenase (COLase) and hyaluronidase (HAase) on in vitro‐cultured neonatal (3‐day‐old and 1‐week‐old) ovaries. The concentration and duration of enzymes were optimized to ensure tissue integrity and ovarian cell viability, as confirmed by H&E staining [13, 14]. Notably, in 3‐day‐old mouse ovaries, ECM components were predominantly localized to the medullary region, while 1‐week‐old ovaries exhibited a more extensive ECM network that permeated the entire ovarian tissue, including the cortical region where primordial follicles are enriched (Figure 3C). One‐week‐old ovaries contained higher collagen content compared to 3‐day‐old counterparts (Figure 3D). Additionally, the ECM in 3‐day‐old ovaries was structurally fragile and more susceptible to enzymatic degradation—even low concentrations of COLase/HAase led to overt tissue fragmentation and loss of follicle integrity (Figure 3E). Therefore, we prioritized 1‐week‐old ovaries for subsequent functional experiments. Compared to the vehicle‐treated control group, COLase‐ and HAase‐treated ovaries exhibited a 20% increase in primordial follicle oocyte diameter (Figure 3F–G). Additionally, enzymatic treatment enhanced nuclear export of FOXO3a in primordial and primary follicles and increased the percentage of proliferating granulosa cells (GCs; Figure 3H–K), collectively indicating accelerated primordial‐primary follicle transition. These results confirm that loosening of ECM structure via degradation of collagen and hyaluronic acid is sufficient to promote primordial follicle activation.

Together, these data demonstrate that targeted degradation of ECM components accelerates primordial follicle activation, and matrisome physical loosening is an instructive cue for follicle awakening. Furthermore, EFD involves nanoscale topographical changes and stage‐specific matrisome patterning, with active ECM remodeling occurring independently of bulk stiffness alterations. These findings integrate structural, functional, and biophysical insights to clarify matrisome's regulatory role in EFD.

### 2.3 Identification of Integrin αvβ3 as the Key Matrisome Sensor on Follicles

Given our prior findings that matrisome composition, structural dynamics, and biophysical properties are dynamically regulated and functionally linked to EFD, we next aimed to identify the cellular surface mediator that transduces matrisome signals into follicles. Integrins are well‐established transmembrane receptors that bridge ECM components with intracellular signaling pathways, making them prime candidates for this role.

**FIGURE 4:**
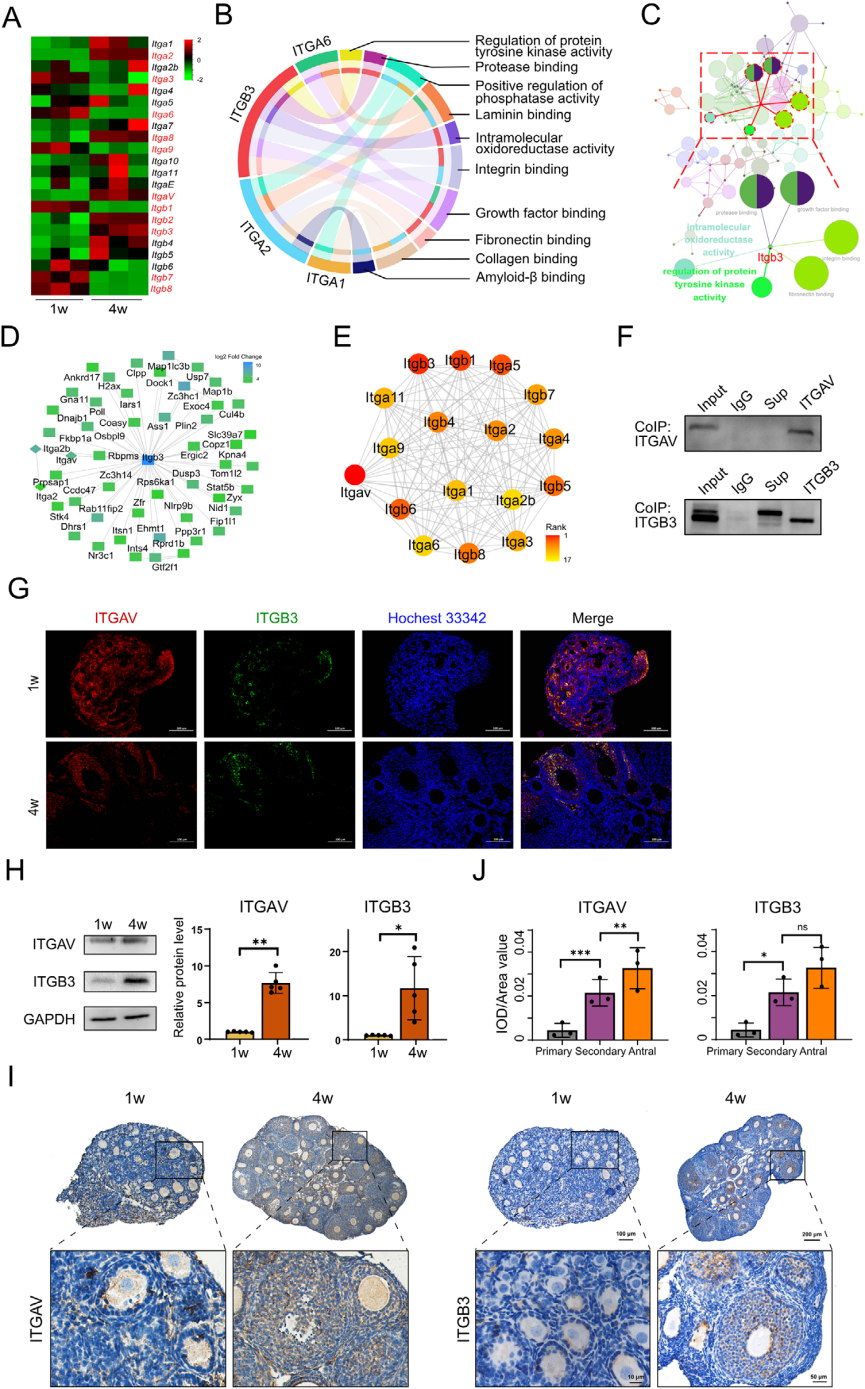
Identification of αvβ3 in Mediating Matrisome‐follicle Crosstalk (A) Heatmap of integrin α and β subunit mRNA expression in 1‐week‐old vs. 4‐week‐old mouse ovaries. (B) Functional association network of integrin DEPs and their related pathways. (C) Protein‐protein interaction conducted through cytoHubba revealed the centered position of *Itgb3*. (D) Co‐immunoprecipitation of ITGB3 followed by LC‐MS/MS identification of interacting proteins. (E) Hub gene network of ITGAV‐interacting proteins (from Co‐IP‐MS), with ITGB3 ranked as the top interacting partner (confidence score = 0.98). (F) Reciprocal Co‐IP validation of ITGAV‐ITGB3 interaction. (G) Dual immunofluorescence staining of ITGAV (red) and ITGB3 (green) in murine ovaries. (H) Western blotting quantification of ITGAV and ITGB3 protein levels in 1‐week‐old vs. 4‐week‐old ovaries (*n* = 5). (I) Immunohistochemical staining of ITGAV and ITGB3 in ovaries from 1 and 4w old mice. (J) Quantitative analysis for ITGAV and ITGB3 staining in primary, secondary, and antral follicles (*n* = 3). Data are presented as mean ± SD. Statistical analysis was performed using Student's *t* test (H, J). All tests were two tailed. *, *p* < 0.05; **, *p* < 0.01; ***, *p* < 0.001; ns, not significant.

To this end, we first profiled the expression of integrin α and β subunits during EFD, as these subunits form functional heterodimers to mediate ECM‐cell interactions. Multiple α subunits (*Itga2*, *Itga3*, *Itga6*, *Itga8*, *Itga9*, and *Itgav*) and β subunits (*Itgb1*, *Itgb2*, *Itgb3*, *Itgb4*, *Itgb7*, and *Itgb8*) showed altered expression (Figure 4A), but proteomic analysis revealed ITGB3 as the only subunit with significant differential expression during EFD (Table S2). Functional annotation and interaction network analysis further supported Itgb3's potential as a signal transducer. It was directly linked to matrisome‐related functional terms, including fibronectin binding, growth factor binding, integrin binding, and protease binding (Figure 4B–C). This aligns with its known role in bridging ECM ligands to cellular responses. These data collectively identified Itgb3 as a promising candidate mediator of matrisome‐follicle communication.

To determine the specific α subunit that pairs with Itgb3 to form a functional integrin heterodimer, we performed co‐immunoprecipitation (Co‐IP) combined with mass spectrometry. This analysis identified 49 Itgb3‐associated proteins, including three α subunits (ITGAV, ITGA2, and ITGA2B; Figure 4D). Notably when ITGAV was used as the bait protein, ITGB3 was among the top interacting partners (Figure 4E). Reciprocal Co‐IP experiments further confirmed the physical interaction between ITGAV and ITGB3 (Figure 4F). Moreover, immunofluorescence co‐localization assays revealed prominent overlapping fluorescent signals of ITGAV and ITGB3 on the cell membrane, providing direct visual evidence of their spatial coexistence (Figure 4G). These validations confirm the formation of the αvβ3 integrin heterodimer. Consistent with a functional role in EFD, both ITGAV and ITGB3 exhibited coordinated upregulation during follicular development Figure 4H–J). These results establish integrin αvβ3 as the key transmembrane mediator that links matrisome signals to early follicles. They also provide a molecular mechanism for ECM‐cell communication in EFD.

### 2.4 Integrin αvβ3 Gatekeeps Primordial Follicle Reserve Through Dose‐dependent Regulation of Activation and Atresia

**FIGURE 5:**
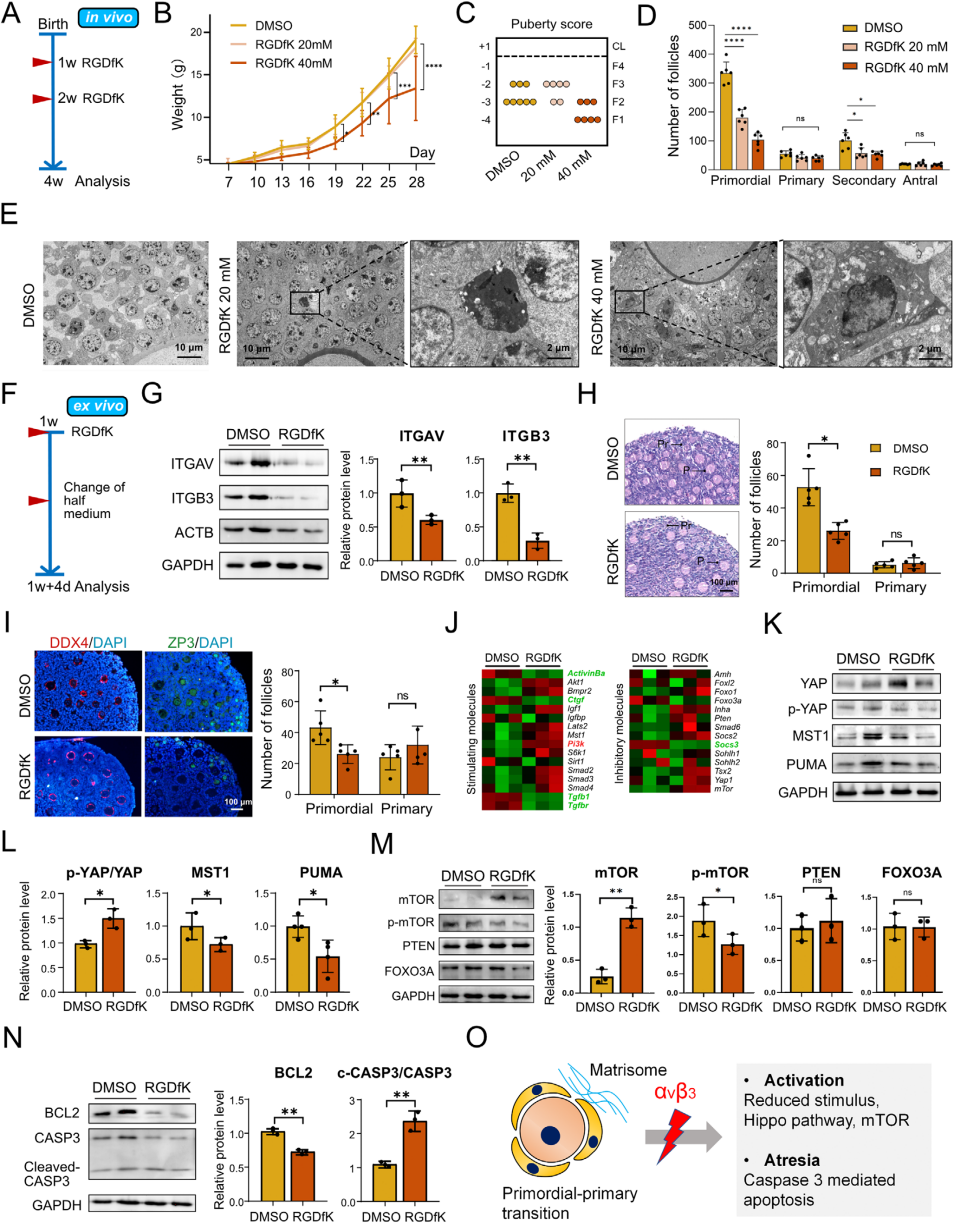
Inhibition of αvβ3 Promotes Primordial Follicle Activation (A) Schematic workflow of in vivo αvβ3 inhibition experiment: neonatal C57BL/6J mice (postnatal day 7) were randomly divided into three groups (DMSO control, 20 µM RGDfK, 40 µM RGDfK), with intraperitoneal injection administered once at 1 week and 2 weeks of age. Mice were euthanized at 4 weeks of age, and ovaries were collected for subsequent analysis (*n* = 10 per group). (B) Body weight changes of mice in each group from 1 to 4 weeks of age (*n* = 6). (C) Ovarian puberty score in 4‐week‐old mice (*n* = 6‐8). (D) Follicle counting in 4‐week‐old ovaries via H&E staining (*n* = 6 mice per group). Follicles were classified according to Pedersen and Peters’ criteria. (E) Transmission electron micrographs images of ovarian follicles in each group, showing apoptotic bodies (red arrows) in RGDfK‐treated groups. (F) Schematic workflow of in vitro αvβ3 inhibition experiment: Neonatal mouse ovaries (postnatal day 7) were cultured ex vivo with 20 µM RGDfK or DMSO control for 4 days, with medium changed every other day (*n* = 5 per group). (G) Western blotting validation of ITGAV and ITGB3 protein downregulation in RGDfK‐treated neonatal ovaries (*n* = 3). (H‐I) Follicle quantification via H&E and DDX4/ZP3 dual‐immunofluorescence (*n* = 5) staining revealed the reduced primordial follicle pool. (J) RT‐PCR analysis of primordial follicle activation‐related genes: stimulating factors and inhibitory factors in RGDfK‐treated vs. control ovaries (*n* = 3). (K‐N) Protein levels of the Hippo signaling pathway (*n* = 3), markers of primordial follicle activation (*n* = 3), and apoptotic markers (*n* = 3). (O) Schematic illustrating the mechanism of αvβ3‐mediated matrisome signaling in primordial follicles. Data are presented as mean ± SD. Statistical analysis was performed using Student's *t* test (G, H, I, L, M, N), one‐way ANOVA (D) or two‐way ANOVA (B). All tests were two tailed. *, *p* < 0.05; **, *p* < 0.01; ***, *p* < 0.001; ****, *p* < 0.0001; ns, not significant.

To delineate the functional role of integrin αvβ3 in primordial follicle development, we treated 1‐week‐old mice in vivo with low (20 mM) and high (40 mM) doses of RGDfK, a specific inhibitor of integrin αvβ3 (Figure 5A). Organ indexes of the heart, kidney, liver, uterus and ovary were comparable across all groups. Mice in the 40 mM RGDfK group exhibited significant weight loss, indicating overt toxic effects of high‐dose inhibition (Figure 5B, Figure S4A–B). We assessed peripubertal ovarian maturation using the puberty score, a reliable histological method for evaluating follicular development timing [15]. The 20 mM RGDfK group showed a higher puberty score than both the control and 40 mM groups, consistent with precocious follicle growth under mild αvβ3 inhibition (Figure 5C). The 40 mM group displayed no such increase, which correlated with substantial atresia across all follicular stages (Figure 5D, Figure S4C). Electron microscopy further confirmed this dose‐dependent effect: apoptotic bodies were detected in both RGDfK‐treated groups, but the control group exhibited predominantly healthy follicles (Figure 5E). These in vivo data demonstrate that low‐dose RGDfK promotes EFD while high‐dose RGDfK impedes follicular growth and induces toxicity

To eliminate systemic confounding factors and directly assess αvβ3's role in follicle‐matrix interactions, we transitioned to an ex vivo culture system [16]. One‐week‐old ovaries treated with 20 mM RGDfK showed reduced primordial follicles, while the number of primary follicles remained stable (Figure 5F–I). To elucidate the mechanisms underlying primordial follicle loss, we analyzed key molecules governing follicle activation and atresia. At the mRNA level, RGDfK significantly upregulated expression of activation‐promoting genes (*Activinb, Akt1, and Bmpr2*; Figure 5J), with only minor increases in inhibitory molecules (*Inha, Socs2, Mtor*). At the protein level, αvβ3 inhibition specifically modulated two core pathways regulating folliculogenesis. Hippo signaling was attenuated, as evidenced by an increased p‐YAP/YAP ratio and decreased levels of MST1 and PUMA (Figure 5K–L). MTOR was significantly upregulated, while FOXO3a and PTEN remained unchanged (Figure 5M). Concurrently, RGDfK induced apoptosis, reflected by reduced BCL2 levels and an increased cleaved‐CASP3/CASP3 ratio (Figure 5N). Therefore, the stable number of primary follicles likely reflects a dynamic balance between enhanced primordial follicle activation and concurrent atresia.

Collectively, these data establish that integrin αvβ3 is essential for the primordial‐to‐primary follicle transition. Inhibition of αvβ3 promotes primordial follicle activation through modulation of the Hippo and mTOR pathways while inducing apoptosis, ultimately leading to reduced primordial follicle reserves (Figure 5O).

### 2.5 Integrin αvβ3 Impairs Secondary Follicles Integrity and Granulosa Cell Function

**FIGURE 6:**
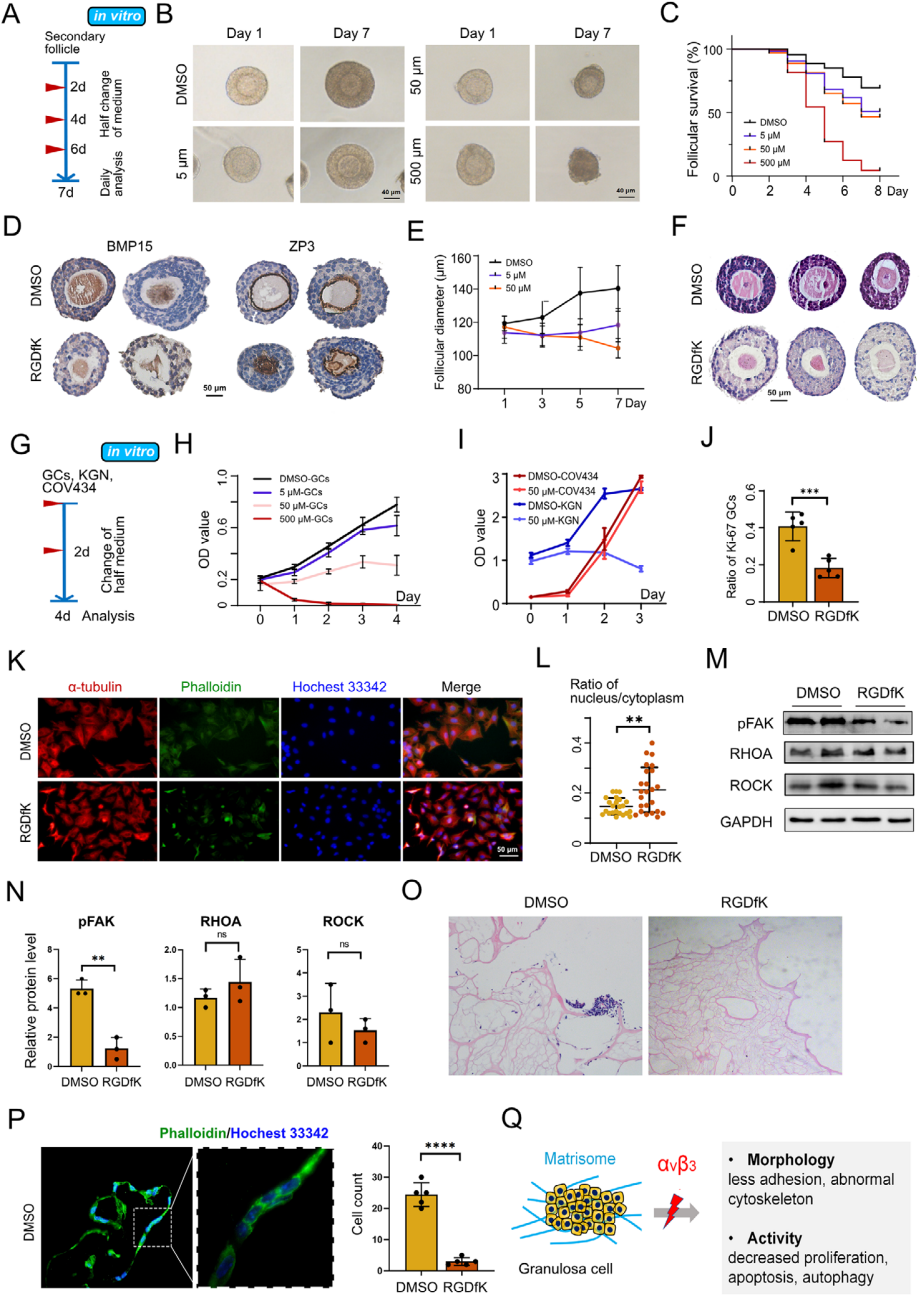
Effects of Disturbing ECM on Secondary Follicles and Granulosa Cells (GCs): (A) Schematic workflow of in vitro αvβ3 inhibition in secondary follicles: Two‐layer secondary follicles were randomly divided into four groups (DMSO control, 5 µM RGDfK, 50 µM RGDfK, 500 µM RGDfK). Medium was changed every other day, and follicle growth was monitored daily (n = 10 follicles per group, three biological replicates). (B) Brightfield images of secondary follicles on Day 1 and Day 7 of culture. (C) Follicle survival rate over 7 days. (D) Immunohistochemical staining of BMP15 (oocyte marker) and ZP3 (zona pellucida marker) in follicles on Day 7. (E) Diameter change over 7 days. (F) H&E staining in follicles on Day 7. (G) Schematic workflow of αvβ3 inhibition in GCs: Primary mouse GCs and human GC‐derived cell lines (KGN, COV434) were cultured in their respective media. Cells were treated with different concentrations of RGDfK (DMSO, 5, 50, 500 µM), and functional assays were performed after 4 days (n = 3 biological replicates per cell type). (H) Cell viability of GCs treated with different concentrations of RGDfK over 4 days, measured by CCK‐8 assay. (I) CCK‐8 assay measuring cell viability of COV434 and KGN cells treated with DMSO or 50 µM RGDfK. (J) quantitative number of primary GCs for Ki‐67 staining. (K) Dual immunofluorescence staining of α‐tubulin (microtubules, red) and phalloidin (microfilaments, green) in primary GCs. (L) Fluorescence intensity and cell volume were quantified (*n* = 24). (M–N) Western blot analysis and quantification of pFAK, RHOA, and ROCK protein levels in cells treated with DMSO or RGDfK. (O) H&E staining of cells cultured on decellularized ECM. (P) Fluorescence imaging of cells on dECM stained with phalloidin (green, for F‐actin) and Hoechst 33342 (blue, for nuclei) in DMSO and RGDfK groups. The bar graph quantifies cell counts in each group. (Q) Illustration described the mechanisms of αvβ3 on GCs. Data are presented as mean ± SD. Data are presented as mean ± SD. Statistical analysis was performed using Student's *t* test (J, L, N, P), or two‐way ANOVA (C, E, H, I). All tests were two tailed. *, *p* < 0.05; **, *p* < 0.01; ***, *p* < 0.001; ****, *p* < 0.0001; ns, not significant.

After identifying αvβ3's role in primordial follicles, we next investigated its function in later folliculogenesis, and secondary follicles were treated with three doses of RGDfK (5, 50, and 500 µM) in vitro (Figure 6A). Follicle survival rates were dose‐dependent, with approximately 50% of follicles survived in the 5 and 50 µM groups, while most follicles in the 500 µM group died within one week (Figure 6B–C). This survival decline correlated with structural damage to oocytes, including disrupted nuclear integrity and zona pellucida (Figure 6D). Follicle growth was also dose‐dependently impaired: control follicles increased in diameter by 17.6%, while 5 µM RGDfK only induced a 4.0% increase, and 50 µM treatment led to reduced follicle size (Figure 6E). This growth retardation was associated with impaired granulosa cell (GC) function, as evidenced by fewer and loosely connected GCs in treated follicles (Figure 6F).

To delineate the direct effects of αvβ3 inhibition on GCs, we treated primary mouse GCs and human GC‐derived cell lines (KGN, COV434) with RGDfK (Figure 6G, Figure S5A). Primary GCs and KGN cells exhibited significant proliferative defects, accompanied by dysregulated expression of cell cycle‐related molecules (Figure 6H–J, Figure S5B–D), while COV434 cells remained unaffected. αvβ3 inhibition also disrupted GC cytoskeleton organization (Figure 6K–L, Figure S5E) and reduced focal adhesion kinase phosphorylation, with no significant impact on the RhoA/ROCK pathway (Figure 6M–N). Consistent with impaired cell adhesion, GCs failed to attach to decellularized ovarian scaffolds after RGDfK treatment, whereas control GCs adhered and maintained normal morphology (Figure 6O–P). Concurrently, RGDfK activated the apoptotic pathway in GCs, as shown by increased pro‐apoptotic markers (*Bax, Bim, Casp3, Casp9*) and decreased anti‐apoptotic *Bcl2* (Figure S5F–G). In contrast, autophagy, pyroptosis, and ferroptosis pathways were not overtly affected, and only three ECM‐related genes (*Mmp1, Mmp23, Tgfb3*) showed significant changes (Figure S5H–I), indicating limited impact on ECM turnover.

Collectively, these data demonstrate that αvβ3 inhibition damages secondary follicles by directly impairing GC function. The underlying mechanisms involve disrupted GC proliferation, cytoskeletal organization, and cell apoptosis, ultimately leading to compromised follicle survival, growth, and structural integrity (Figure 6Q).

### 2.6 αvβ3 inhibition Directly Impairs Oocyte Maturation

**FIGURE 7:**
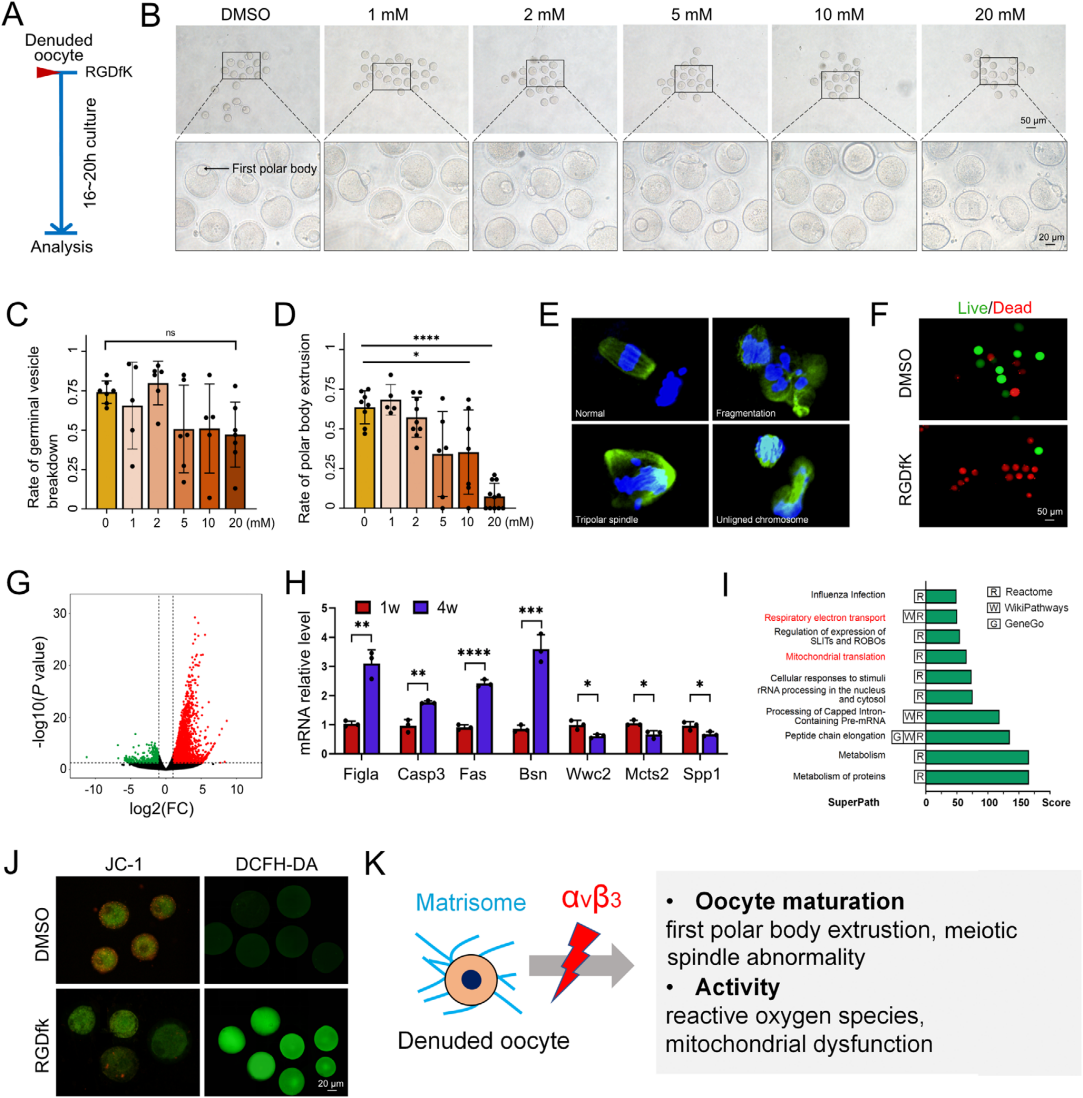
Effects of Integrin Inhibition on Denuded Oocytes: (A) Schematic workflow of αvβ3 inhibition in denuded oocytes: Denuded germinal vesicle oocytes were treated with different concentrations of RGDfK (DMSO, 1, 2, 5, 10, and 20 µM) for 16–20 h. (*n* = 20 oocytes per group, three biological replicates). (B‐D) Representative images and statistics for rates of germinal vesicle breakdown (GVBD) and polar body extrusion (PBE) at different concentrations. (E) Immunofluorescence staining of α‐tubulin (spindle, green) and DAPI (chromosomes, blue) in MII oocytes. (F) Live‐dead staining images of oocytes after 12 h culture in RGDfK. Green: live cells; red: dead cells. (G) Volcano map of DEGs in MII oocytes using SMART‐Seq2 method. (H) Validation of the DEGs in SMART‐Seq2 data (n = 3). (I) Integrated pathway enrichment analysis of DEGs using databases including Reactome, WikiPathways, and GeneCards. (J) Reactive oxygen level of MII oocytes stained with JC‐1 and DCFH‐DA probes. (K) Illustration described the mechanisms of αvβ3 on secondary follicles. Statistical analysis was performed using Student's t test (H) or two‐way ANOVA (C, D). All tests were two tailed. *, *p* < 0.05; **, *p* < 0.01; ***, *p* < 0.001; ****, *p* < 0.0001; ns, not significant.

Upon completing our characterization of αvβ3 in early follicles and granulosa cells, we finally focused on the oocyte, the core functional unit of the follicle (Figure 7A). To isolate direct effects on oocytes, we treated denuded oocytes with increasing concentrations of the αvβ3 inhibitor RGDfK (1, 2, 5, 10, and 20 nM) and assessed maturation outcomes. While germinal vesicle breakdown was comparable across all RGDfK doses, the rate of first polar body extrusion decreased in a concentration‐dependent manner: 20 nM RGDfK reduced extrusion rates to nearly 0, whereas intermediate doses (5–10 nM) caused partial impairment (Figure 7B–D). This maturation failure was accompanied by overt meiotic defects. Treated oocytes exhibited DNA fragmentation, tripolar spindle formation, and chromosome misalignment (Figure 7E), which ultimately led to oocyte aneuploidy and death (Figure 7F).

To dissect the molecular mechanisms underlying these defects, we performed SMART‐Seq2 transcriptomic profiling of RGDfK‐treated versus DMSO‐control oocytes. This analysis identified 532 DEGs (Figure 7G), with expression trends of key candidates (*e.g., Figla, Casp3, Fas*) validated to confirm sequencing robustness (Figure 7H). Notably, the expression of *Figla*, a key regulator of oocyte development, was significantly upregulated, indicating profound alterations in the meiotic process. The increased expression of *Casp3* and *Fas* suggested the activation of apoptotic pathways following αvβ3 inhibition. Conversely, *Wws2* and *Spp1*, genes implicated in the Hippo pathway and ECM binding, respectively, showed a decreasing trend, further supporting the role of αvβ3 in ECM‐mediated signaling. Gene Ontology analysis revealed that the majority of DEGs were associated with oocyte metabolism and mitochondrial function, including protein metabolism, mitochondrial translation, and respiratory electron transport (Figure 7I). Consistent with these findings, mitochondrial dysfunction and elevated reactive oxygen species levels were confirmed by JC‐1 and DCFH‐DA staining, respectively (Figure 7J). Collectively, these results demonstrate that αvβ3 inhibition disrupts core oocyte functions (meiosis, metabolism, viability) through dysregulated apoptosis and mitochondrial homeostasis (Figure 7K).

### 2.7 Conservation of Matrisome‐αvβ3 Dynamics in human Ovaries Underscores Translational Relevance

**FIGURE 8:**
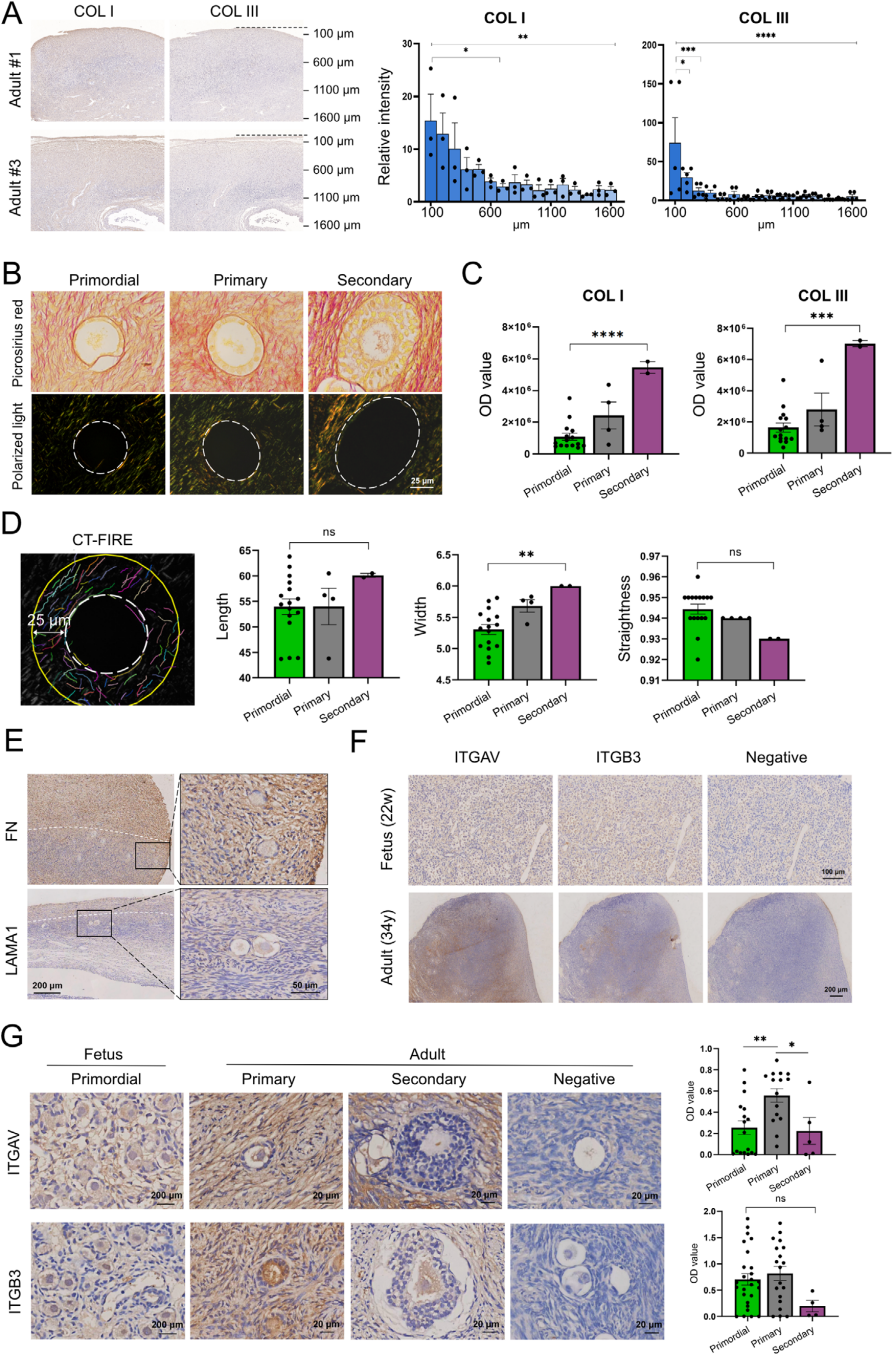
Changes in Collagen and αvβ3 on human Ovarian Tissues: (A) Immunohistochemical staining of collagen I (COL I) and collagen III (COL III) in adult (22–36 years old, *n* = 9 patients) human ovaries at different depths from the surface. (B) Brightfield and polarized light images of Sirius red‐stained collagen fibers surrounding human follicles (<25 µm in diameter) at primordial, primary, and secondary stages. (C) Quantification of COL I and COL III content (OD value at 540 nm) surrounding follicles. Data were collected from five individuals. (D) Length, width, and straightness were compared between various developmental stages (five biological replicates each group). (E) Immunohistochemical staining of ECM proteins. (F) Immunohistochemical staining of ITGAV and ITGB3 in fetal (22 weeks) and adult (36 years) human ovaries. (G) Quantification of ITGAV and ITGB3 expression in primordial, primary, and secondary follicles via ImageJ. Data are presented as mean ± SD. Statistical analysis was performed using one‐way ANOVA (A, C, D, G). All tests were two tailed. *, *p* < 0.05; **, *p* < 0.01; ***, *p* < 0.001; ****, *p* < 0.0001; ns, not significant.

To bridge our findings to human physiology, we analyzed adult ovaries (22–36 years) to characterize matrisome changes during folliculogenesis, and supplemented with fetal ovaries (22–34 weeks) to assess integrin expression across developmental stages. In adult ovaries, we prioritized collagen analysis given its established role in modulating ovarian mechanical properties. The levels of fibrillar collagens (COL1, COL3) were highest in the ovarian cortex and decreased toward the medulla (Figure 8A), consistent with prior reports of cortical‐medullary stiffness gradients [17]. Around follicles (<25 µm in diameter [18]), COL1 and COL3 levels increased with follicular growth (Figure 8B–C). Extraction of follicle‐associated collagen fibers revealed no difference in fiber length across primordial, primary, and secondary follicles, but advanced‐stage follicles exhibited wider fibers, likely due to increased cross‐linking (Figure 8D). Additionally, regions with sparse FN1/LAMA1 staining contained more follicles (Figure 8E), aligning with our mouse ECM proteolysis data showing dense matrisome restricts follicle activation. Regarding integrin expression, both ITGAV and ITGB3 subunits were widely expressed in fetal and adult ovaries (Figure 8F). Their levels peaked in primary follicles, while ITGB3 expression remained stable across follicular stages (Figure 8G). These human data confirm the conserved principles of stage‐specific matrisome remodeling and αvβ3 expression, unifying our cross‐species observations and highlighting the translational relevance of this mechano‐signaling axis in human folliculogenesis.

## 3 Discussion

The EFD establishes the foundation for female fertility, yet the regulatory role of the ECM microenvironment, the matrisome, remains poorly defined. Here, by integrating multi‐omics, nanomechanical mapping, and functional perturbations, we delineate a dynamic matrisome landscape throughout EFD and identify integrin αvβ3 as a crucial mechano‐chemical sensor transducing ECM signals into follicular fate decisions. The study highlights the crucial role of matrisome in regulating EFD and provides novel insights into addressing ovarian dysfunction and advancing assisted reproductive technologies.

The ovarian matrisome, a complex assembly of ECM components, is integral to the processes of folliculogenesis and oocyte maturation. These proteins are essential for maintaining the ECM that surrounds the follicles, providing both mechanical support and biochemical signals necessary for follicular development and maturation. Studies have shown that matrisome may influence the localization and development of follicles. For instance, in bovine ovaries, a greater concentration of follicles were found near the ovarian pedicle compared to the greater curvature of the ovary [19]. Changes in the rigidity of the ovarian ECM affect follicle activation, with a stiffer ECM being less permissive to follicle growth [20]. Furthermore, the matrisome may even facilitate cancer progression, indicating a broader function for the matrisome in cellular metabolism [21].

Our study focused on constructing a map of ovarian matrisome, with a particular emphasis on observing the structural alterations during EFD. AFM is conventionally used to display the outer surface of an object. It operates by scanning a sharp probe over the surface of a sample to generate detailed 3D images of the surface structure [22]. Our observation that ovarian morphology becomes increasing rough during EFD suggests significant changes in terms of volume expansion and ECM remodeling. This phenomenon is corroborated by several studies exploring the dynamics of follicular growth and ECM changes, which demonstrate that both collagen and elastin change with age and follicle stage, thereby impacting follicle activation and development [20]. Moreover, the application of collagenase for ovarian disaggregation also highlights the critical role of ECM components in maintaining follicle structure and viability [23]. ECM degradation surrounding preovulatory follicles reduces mechanical stress and triggers the activation of adjacent primordial follicles [24].

Developing systematic ovarian matrisome atlases is being acknowledged as a transformative technique that surpasses descriptive analysis. First, high‐resolution and multi‐omics maps reveal the exact relationship between ECM alterations and follicle outcomes from the basic science view. And the CLARITY technique has been employed to better to exhibit the details of ovarian structure and blood vessels [25]. In terms of bioengineering, the matrisome can serve as a reference for ovarian scaffolds and then, together with isolated follicles, form a biocompatible artificial ovary [26, 27, 28]. Lastly, by elucidating the complex dynamics of ECM remodeling, these atlases can inform the design of targeted therapies that address ECM dysregulation. Hydrogels and other ECM‐derived materials can provide spatial and temporal control over drug release, facilitating cell‐targeted delivery and improving therapeutic outcomes [29].

While decellularization is a powerful technique for isolating and analyzing the ECM scaffold, we opted not to employ it for investigating the matrisome in the context of ovarian follicular development. One key reason is that the decellularization process itself, involving detergent treatments and mechanical agitation, can lead to the loss or modification of labile ECM components [30, 31]. For instance, SDS is known to diminish tissue mechanical strength through removal of structural proteins and glycosaminoglycans [32], and trypsin can significantly degrade collagen and other ECM proteins [33]. Instead, we utilized a combination of transcriptomic, proteomic, and functional assays in intact ovarian tissues and isolated follicles to capture the matrisome in its physiologically relevant, cell‐associated state, allowing us to study the matrisome's role within the context of the complex cellular microenvironment that drives follicular development.

Integrins are main receptors in mediating matrisome‐cell interaction. Although αvβ3's function and mechanism in cancers have been broadly investigated [34], less is known about the role of it in ovaries. ITGB3 was reported to increase in cultured GCs and possibly regulates cell growth and angiogenesis together with ITGAV [35]. Another study observed an increased expression of αvβ3 in atretic GCs, and an ITGB5‐ITGB3‐CAV2 network might regulate oocyte development and maturation [36]. Therapeutics that target integrins are potential treatment for a series of diseases. For example, the αvβ3/αvβ5 antagonist cilengitide has been studied on head and neck squamous cell carcinoma [37]. Abituzumab, an antibody targeting αv integrins, was tested in inhibiting the migration and invasion in prostate cancer [38]. This research for the first time regulated αvβ3 expression to investigate its role in EFD, which may be a potential target for matrisome related disturbance in ovaries.

The Hippo pathway is highly conserved among species and is regulated by both intracellular and extracellular signals [39]. Activation of the Hippo pathway in GCs is well known to inhibit the development of primordial follicles [40]. Conversely, the inhibition of the Hippo signaling pathway promotes primordial follicle activation, which is the mechanism underlying in vitro activation and has led to successful delivery [41]. Moreover, the activated Hippo signaling pathway controls size enlargement in liver, heart and other organs [42]. However, the ovarian volumes decrease after integrin intervene. It may be resulted from the massive death of follicles. Ovarian fragmentation is suggested to cause actin polymerization, followed by p‐YAP dephosphorylation and Hippo pathway disruption [43]. Our results support a potential link between αvβ3 and actin dynamics, which aligns with established literature showing that αvβ3 mediates actin cytoskeleton remodeling in osteoclasts, endothelial cells, and tumor cells [44, 45, 46].

Matrisome proteins are first synthesized within the intracellular endoplasmic reticulum and Golgi apparatus before undergoing secretion and subsequent assembly into complex, functional extracellular networks outside cells. As such, genetic perturbation inevitably disrupts both the intracellular and extracellular pools, often complicating the interpretation of specific ECM functions [47, 48]. Compounding this issue is the inherent complexity of the ECM itself: the ovarian matrisome operates not as a collection of isolated, functionally independent molecules, but as an integrated supramolecular system where collagens, glycoproteins, proteoglycans, and ECM regulators interact dynamically to maintain tissue structure and transmit biochemical signals [49, 50]. Within this system, functional redundancy is prevalent. For instance, multiple collagen subtypes may share overlapping binding sites with integrin receptors [51, 52]. Against these backdrops, enzymatic proteolysis was driven by a critical need to specifically target the extracellular ECM architecture, while minimizing unintended intracellular disruption [13, 14]. Unlike genetic approaches, these enzymes act exclusively within the extracellular space, allowing us to isolate the effects of ECM structural perturbation from confounding intracellular events.

This study has some limitations. The dynamic ECM changes during the primary‐to‐secondary follicle transition warrant finer temporal resolution. The contribution of other integrins beside αvβ3 also merits exploration. Most importantly, while our human tissue data show conserved patterns of matrisome remodeling and αvβ3 expression, ethical and practical constraints prevent functional validation in human ovaries. Future work employing human ovarian organoids or xenograft models, alongside cell‐type‐specific manipulation strategies, will be vital to translate these mechanisms directly to human reproductive health.

## 4 Conclusion

In conclusion, we provide a comprehensive map of the ovarian matrisome as a dynamically remodeled, instructive niche. By identifying integrin αvβ3 as a key sensor that translates ECM dynamics into balanced follicular growth and survival status, we establish a new mechanistic framework for EFD. This work not only advances our understanding of ovarian biology but also opens concrete avenues for innovative strategies in fertility preservation and the treatment of ovarian disorders.

## 5 Experimental Section

### 5.1 Experimental Design and Animal Group

Female C57BL/6J mice and KM mice were obtained from the center for disease control and prevention of Hubei Province in Wuhan. Animals were randomly housed in a controlled temperature (24°C) and subjected to 12 h light–dark cycles, with free access to diet and water ad libitum. KM mice were only utilized to isolate denuded oocytes and other experiments all used C57BL/6J mice. Each experiment was conducted with at least three replicates.

All experimental protocols involving animal handling were approved by the institutional animal ethics committee of Huazhong University of Science and Technology (ID: 127–20230430) and conducted in accordance with the requirements of the national research council guide.

### 5.2 Antibody Reagents

The primary and secondary antibodies used in immunohistochemical staining, immunofluorescence staining, Western blots, and co‐immunoprecipitation assay experiments in this study were strictly selected based on experimental objectives and target protein characteristics. The antibody information is listed as follows: LC3B (ABclonal, A19665), P62 (ABclonal, 11483), Bcl – 2 (ABclonal, A19693), ITGAV (BOSTER, BM5155), Alexa Fluor488 Donkey anti Rabbit IgG (antgene, ANT024s), Alexa Fluor594 Donkey anti Rabbit IgG (antgene, ANT030), COL1A1 (BOSTER, BA0325), CTGF (BOSTER, BA0752), COL2A1 (BOSTER, BA0533), COL3A1 (BOSTER, PB0125), FN (Servicebio, GB112093), TGFB1 (Servicebio, GB111876), Ki67 (Servicebio, GB11030), Laminin (Novus, NB300‐144), AMH (Proteintech, 14461‐1‐AP), C3 (Proteintech, 21337‐1‐AP), CD18 (Proteintech, 10544‐1‐AP), CD14 (Proteintech, 17000‐1‐AP), H2AB1 (ABclonal, A18658).

### 5.3 Quantification of Ovarian Follicles

Fresh ovaries were fixed overnight at room temperature in 4% paraformaldehyde, dehydrated through a gradient of ethanol, clarified with xylene, and then embedded in paraffin. Inclusion blocks were then serially sectioned at a thickness of 5 µm. In hematoxylin and eosin (H&E) approach, sections of 80 µm were selected and stained with H&E solution. Ovarian follicles of different stages were classified as follows: Primordial follicles consist of a single oocyte surrounded by one or more layers of flattened pre‐granulosa cells. Primary follicles have a single oocyte encircled by a mixed layer of cuboidal or squamous granulosa cells. Secondary follicles feature an enlarged oocyte encased by several layers of cuboidal granulosa cells, as well as an antral follicle. Follicles were included only if the oocytes had a visible nucleus. [53].

MVH and ZP3 were used as immunofluorescent indicators. MVH is a specific oocyte marker, and we use MVH positive oocyte counts to represent the total number of follicles. ZP3 is expressed in the zona pellucida in growing follicles, but not in primordial follicles. Therefore, we use the number of ZP3 positive oocytes to represent the number of growing follicles. During the culture of neonatal ovaries, the growing follicles mainly refers to primary follicles. The number of primary follicles is the difference between MVH and ZP3.

### 5.4 Immunofluorescence (IF)

Following dewaxing and rehydration, the sections underwent heat‐induced antigen retrieval by boiling them in a 10 mM sodium citrate buffer for 10 min. Afterward, the sections were blocked using 5% bovine serum albumin (BSA) for 30 min at room temperature and then incubated with either primary antibodies or PBS (as a negative control) at 4°C overnight. On the next day, the sections were incubated with donkey anti‐rabbit Alexa Fluor 488‐ or 594‐conjugated secondary antibodies for 1 h at 37°C, and subsequently stained with 4′,6‐diamidino‐2‐phenylindole (DAPI) for 10 min. Eventually, sections were examined using a fluorescence microscope (BX53, Olympus, Japan)

### 5.5 Estrous Cyclicity Examining

To define the research timespan, a total of 20 C57BL/6J mice were adopted to examine the vaginal opening and estrus stages. Vaginal smear was obtained daily by gentle lavage for three times using 30 µL normal saline. Allow the sections to air dry, perform H&E staining and observe under bright‐field microscopy. The stage of the estrous cycle was determined by examining the cellular morphology and composition.

### 5.6 Hormone Assay

Five female C57BL/6J mice aged from weeks three to five in each group were sacrificed. Blood samples were collected via the eyelid venous under anesthesia. Samples coagulated for 1 h at room temperature before being centrifuged at 3000 rpm for 20 min. Serum samples were preserved at –80°C before analysis, and estradiol, progesterone, and FSH were measured with ELISA kits. Estradiol ELISA Kit (CSB‐E05109m, Cusabio, China), Progesterone ELISA Kit (CSB‐E05104m), FSH ELISA Kit (CSB‐E06871m), according to the manufacturers’ instructions. The analytical specificity was 100% for all metrics. The coefficient of variation of inter‐assay was less than 4%. The correlation coefficient of the standard curve was 0.9995.

### 5.7 RNA Extraction and Real‐Time Quantitative Polymerase Chain Reaction (RT‐PCR)

Total RNA was extracted using RNAiso Plus solution (9109, Takara, Japan) and the concentration was measured with NanoDrop (ND‐ONE‐W, Thermo Scientific, USA). First strand complementary DNA (cDNA) was converted from RNA (500 ng) using random primers and HiScript II reverse transcriptase (R123‐01, Vazyme, China). For RT‐PCR assays, a reaction mixture of 20 µL containing 10 µL ddH2O, 8.2 µL ChamQ universal SYBR (Q711‐02/03, Vazyme, China), 0.4 µL forward and reverse gene‐specific primers each (Tsingke, China) and 1 µL cDNA template. Three biological replicates and three technical replicates were designed for each indicator.

### 5.8 Bulk RNA Sequencing Analysis

Polyadenylated RNAs of 1‐ and 4‐week ovaries were purified using a NEBNext Poly(A) mRNA Magnetic Isolation Module (E7490S/L, New England Biolabs). Biologically triplicated samples were prepared. Purified RNAs were subjected to library construction using a NEBNext Ultra II mRNA Library Prep Kit for Illumina (E7775, New England Biolabs). The Qubit dsDNA HS Assay Kit was employed to determine the library concentration, and the Agilent 4200 was used to check the segment distribution for quality control. The KAPA Library Quant kit (Illumina) universal qPCR Mix was used to determine the library's molar concentration. High‐throughput transcriptome sequencing was carried out on an Illumina NovaSeq 6000 platform. Limma R was used to obtain differential expressed genes (DEGs) in each dataset with the criteria of |log2(foldchange)| > 1 and p < 0.05.

### 5.9 Functional Enrichment Analysis

Online website GeneAnalytics was used to perform signaling pathway analysis [54]. Pathway analysis through gene set variation analysis focused mainly on hallmark pathways from GSVA Ver.1.22.4. Matrisome gene sets were downloaded from The Molecular Signatures Database website (Version 4.1.0) software to perform gene set enrichment analysis (GSEA). The program repeated 1000 times, and *p* < 0.05. The ClueGO plug‐in in Cytoscape 3.8.1 software was used for molecular function analysis. The species, enrichment method and network specificity were set as Mus Musculus, GO‐molecular function, and medium, respectively. Rotational gene set tests (ROAST) were executed using the roast function within the limma package. Genes were weighted in the gene set tests through the use of moderated t‐statistics. The barcode plot function of the limma package was employed to create barcode plots.

### 5.10 Western Blotting (WB) and Co‐Immunoprecipitation (Co‐IP)

Total protein was obtained from the ovarian tissues with RIPA lysis buffer (G2002, Servicebio, China), added with 1 mM phosphorylase inhibitor and phenylmethylsulfonyl fluoride. The tissues were milled, sonicated, centrifuged and finally heated at 100°C for 10 min. The concentration was measured by a Bradford assay (P0012, Beyotime, China). After separation on a 10% SDS‐PAGE gel, the samples were transferred to PVDF membranes (GVS, Italy) using a wet blotter in transfer buffer at a constant voltage of 270 V for a specific time. The membranes were incubated in 5% BSA for 30 min and subsequently incubated with primary antibodies overnight at 4°C. On the next day, the membranes were incubated with the secondary antibody at 37°C for 1 h. The immunoblots were detected using an enhanced chemiluminescence kit (K‐12045‐D50, Advansta, USA) and visualized using a chemiluminescence imaging system (ChemiDoc, BioRad, USA). The bands were densitometrically quantified using Image lab and normalized against the corresponding intrinsic control.

To perform Co‐IP, IgG or the designated primary antibodies were combined with the protein extract and incubated at 4°C overnight. On the subsequent day, Protein A/G magnetic beads (HY‐K0202, MCE) were added and gently shaken for 2 h at 4°C. After washing the precipitates of each sample five times with a buffer lacking detergent, they were boiled for five minutes to elute and then immunoblotted with the specified antibodies.

### 5.11 Decellularized Mouse Ovary

Five ovaries of each group were utilized for decellularization. The ovaries were frozen in a ‐80°C freezer for 30 min, and then were thawed for another 30 min. The frozen‐thawed cycle was reiterated three times. Subsequent tissues were immersed in double‐distilled water and agitated at 37°C. Following this, the tissues were subjected to a continuous agitation process for four days within a solution comprising a mixture of 2% sodium deoxycholate and 4% Triton X‐100. The washing solution was refreshed every 12 h. Finally, the tissues were treated with a DNA enzyme solution (80 U/mL) at 37°C to remove residual nucleic acid.

### 5.12 Sirius Red Staining and Alcian Blue

The sections were immersed in Sirius red staining solution (GP1033, Servicebio, China) and Alcian blue (GP1040, Servicebio, China) for 30 min, respectively.

### 5.13 Scanning Electron Microscopy (SEM)

The structure of decellularized ovaries was studied with SEM. Samples were freeze‐dried and positioned on objective tables before undergoing gold sputtering. HITACHI Regulus 8100 was used to analyze and photograph the specimens.

### 5.14 Masson's Trichrome

The sections were applied with potassium dichromate (GP1032, Servicebio, China) at 37°C for 4 h. Rinse the sections with tap water for 30 s until the tissue appears colorless. Then treat the sections with the iron hematoxylin staining solution for 3 min, briefly rinse under running water, and then immerse in 1% hydrochloric acid alcohol for 1 min. Immerse the sections in eosin methylene blue reagent, 1% phosphomolybdic acid, and aniline blue for 8 min, 2 minutes, and 8 s, respectively.

### 5.15 Van Gieson Staining

The sections were immersed in Van Gieson solution (GP1141, Servicebio, China) for 2 min, followed by rinsed in 2% ferric chloride solution for 10 s.

### 5.16 Ovary Culture

Neonatal ovaries were collected at postnatal day 7 and dissected carefully to maintain the integrity. Eight to ten ovaries were randomly selected as a group and cultured on a semipermeable membrane (110414, Millipore, USA) in 24‐well culture dish at 37°C and 5% CO_2_. Each well contained 500 µL α‐MEM (Gibco, Life Technologies, USA) supplemented with ITS‐G, 100 mM sodium pyruvate (11360070, Gibco, USA), L‐Ascrobic acid (A5960‐25G, Sigma‐Aldrich, USA), and penicillin‐streptomycin. For ovaries treated with RGDfK, dimethylsulfoxide (DMSO) was used as a control. After seven days of culture, the ovaries were collected. FITC‐RGD was purchased from Frdbio Co., Ltd in Wuhan, China.

### 5.17 Immunohistochemistry (IHC)

IHC was performed according to the ready‐to‐use SABC kit (SA1052, Boster, China). Endogenous peroxidase activity was neutralized for 30 min at room temperature using 3% hydrogen peroxide. Sections were blocked with 5% BSA for half an hour and then incubated overnight at 4°C with primary antibodies or PBS, which served as a negative control. Sections were incubated the next day with a biotin‐conjugated goat anti‐rabbit IgG secondary antibody for an hour, and then with SABC reagents for 30 min. For avidin peroxidase detection, the diaminobenzidine solution (DAB, AR1027, Boster) was applied, followed by counterstaining the sections with hematoxylin. Sections were examined using a fluorescence microscope (BX53, Olympus, Japan). At least three slides were used for every index.

### 5.18 Matrisome Component Annotation

Matrisome components in mouse ovaries were annotated using MatrisomeDB (v2.0) [8], a comprehensive ECM‐protein database, following standardized workflows [55]. Gene symbols and UniProt IDs were standardized to MatrisomeDB identifiers using the org.Mm.eg.db R package. Mapped genes/proteins were categorized per MatrisomeDB annotations, and unmapped entries were labeled “non‐matrisome”. Annotated components were visualized using differential expression heatmaps. Representative components were localized via IHC, confirming spatial consistency with MatrisomeDB annotations.

### 5.19 Atomic Force Microscope (AFM) Indentation Measurements

Using a commercial setup (NT‐AIST, HORIBA, Japan), AFM measurements were carried out in force mapping mode with a high‐quality tip that has a diameter of under 8 nm (MikroMasch, USA). The spring constant of the cantilever was 0.5 N. The topography and Young's modulus image were recorded to identify the ovarian structures (*n* = 5 each group). Each ovary was mapped in a 50 × 50 µm square grid with an integration space at 5 µm. Sa was calculated using the following formula:

$$\mathrm{Sa}=\frac{1}{N}\sqrt{\sum_{i=1}^{N}{\left(Z_{i}-Z_{avg}\right)}^{2}}$$

Here, *N* indicates the number of data points for surface height, *Z_i_* is the height value at the *i*th point, and *Z_avg_* stands for the average surface height.

### 5.20 Raman Mapping

Confocal Raman microscopy (HORIBA HR Evolution) was used to capture Raman images of ovarian samples, utilizing a laser with a 532 nm excitation wavelength. The images were collected through a 50× air objective (Zeiss Plan‐Neofluar, NA 0.90) with a laser power of 1 mW. The ovarian samples were scanned over an area of 67 µm × 49 µm with *x* and *y*‐direction steps of 1 µm and an integration time of 2 s per spectrum.

### 5.21 Non‐negative Matrix Factorization Analysis

Data analysis was conducted using an in‐house written script based on Gnu R, and the NNLM package. The Raman datasets from ovarian sample images underwent initial preprocessing through multiple steps to eliminate the detrimental influences present in the measured Raman spectra.

The Raman spectra were first shortened to the wavenumber range of 400–2000 cm^−1^. Following this, cosmic spikes were eliminated by comparing each set of Raman spectra. SNIP was employed for baseline correction, followed by a 9‐point Savitzky–Golay smoothing to reduce noise. Finally, area normalization was conducted on all spectra to correct for spectral variations resulting from changes in focus conditions.

Once preprocessing was complete, the Raman spectra were subjected to analysis via the non‐negative matrix factorization method to map the spatial distribution of the relative contents of various components. With this method, the spectral matrix A_n × m_ was decomposed into two non‐nagative matrixes W_n × k_ and S_k × m_. Matrix W_n × k_ consists of spectra of “pure” chemical component in ovarian samples, while S_k × m_ represents the concentration profiles corresponding to each pure component.

### 5.22 Determination of Matrisome Atlas

ECM proteins, including six categories of core ECM (collagen, proteoglycan, and glycoprotein) and ECM‐associated proteins (ECM regulator, ECM‐affiliated protein, and secreted factor), were selected based on the previously published in silico characterization of the murine matrisome [56]. We identified 228 matrisome genes to study and compare their contents.

### 5.23 Liquid Chromatography‐Tandem Mass Spectrometry (LC‐MS/MS)

LC‐MS/MS data acquisition was carried out on a Q Exactive HF mass spectrometer coupled with UltiMate 3000 RSLCnano system. Peptides were loaded through auto‐sampler and separated in a C18 analytical column. The separation gradient was established using mobile phase A, which contained 0.1% formic acid, and mobile phase B, which consisted of 80% ACN and 0.1% formic acid, with a steady flow rate of 300 nL/min. Each scan cycle in DDA mode analysis comprises one full‐scan mass spectrum and 20 MS/MS events.

Using the Andromeda database search algorithm, the raw data were examined. The spectra files were compared to the Mouse database (2023‐01‐03, 17132 entries) in Uniprot with the specified parameters. Search results were refined using a 1% FDR threshold at both the protein and peptide levels. Interactors of the bait protein were identified by screening proteins with a fold change exceeding 4 and a *p* value below 0.05 between bait IP and controls.

### 5.24 ECM Proteolysis

To investigate the effect of ECM degradation on early follicles, we treated neonatal mouse ovaries with collagenase type IV (COLase) or hyaluronidase (HAase) (n = 5 each group with three replicates). COLase solution

Prepared in α‐MEM medium (supplemented with 3% BSA, 10 mIU/mL recombinant FSH, 5 µg/mL ITS‐G, and 0.5% penicillin‐streptomycin to a final concentration of 0.1 mg/mL collagenase type IV. 1 µM CaCl_2_ and 0.025% trypsin EDTA were added to enhance enzyme activity. HAase solution: Prepared in α‐MEM medium (with the same supplements as above) to a final concentration of 100 µg/mL hyaluronidase. Upon enzyme treatment, the medium was replaced with 500 µL of COLase or HAase solution and ovaries were cultured at 37°C with 5% CO_2_ for 6 h to ensure ECM degradation. H&E staining was used post‐enzyme treatment to confirm that the tissue structure was intact and ovarian cells were alive.

The collagenase type IV solution was prepared as 1 µM CaCl2, 0.1 mg/ml collagenase type IV, 20% BSA, and 0.025% trypsin EDTA, according to previous studies [13, 14]. Hyaluronidase was prepared as 100 µg/mL in PBS. Postnatal 7 ovaries treated with collagenase or hyaluronidase for 6 h at 37°C and 5% CO2.

### 5.25 Intraperitoneal Injection Treatment

One‐week C57BL/6J mice were randomly divided into the DMSO, RGDfK 20 mM, and RGDfK 40 mM groups (n = 6–8 samples each group). Each mouse received intraperitoneal injection once at 1 week and 2 weeks of age, and their weight was measured every three days. They were euthanized at 4 weeks of age and their organs were collected. The appropriate RGDfK doses were determined by preliminary dose‐screening experiments (n = 3 biological replicates per dose group). We tested RGDfK doses of 5 mM, 10 mM, 20 mM, 40 mM, and 60 mM. Doses ≤10 mM showed no significant effect on primordial follicle activation; 20 mM and 40 mM induced significant primordial follicle transition while maintaining ovarian cell viability; 60 mM caused massive ovarian apoptosis and tissue shrinkage. Thus, 20 mM (low dose) and 40 mM (high dose) were selected for in vivo injection.

### 5.26 Puberty Score (Pub‐Score) Assessment for Pre‐Pubertal Mice [15]

The 10‐µm thick ovarian tissue sections were stained with H&E to observe the morphology of the follicles. The diameter of the six largest healthy antral follicles was measured in the selected sections. Antral follicles were categorized into a baseline group and four discrete classes (F1–F4) based on diameter. Baseline

Small follicles (SF) with diameter <250 µm; F1: 250–300 µm; F2: 301–350 µm; F3: 351–400 µm; F4: >400 µm. After confirming the most advanced healthy antral follicle class in each ovary, a Pub‐score ranging from ‐5 to ‐1 was assigned. SF<250 µm (most immature follicles): Pub‐score = ‐5; F1: Pub‐score = ‐4; F2: Pub‐score = ‐3; F3: Pub‐score = ‐2; F4 (most advanced class before ovulation): Pub‐score = ‐1. This scoring system reflects the progressive maturation of ovarian follicles, where higher Pub‐scores indicate that the ovary is closer to achieving ovulatory competence.

### 5.27 Preantral Follicle Culture

Two‐layer secondary follicles (∼110 µm) were isolated from 14‐day‐old C57BL/6J strain female mice using 30G needles. Only healthy follicles were selected for the following experiments. The healthy follicles were defined as: 1) no pyknotic bodies, pyknotic nuclei or cytoplasmic retraction; 2) surrounded by well‐organized layers of granulosa cells; 3) no signs of degeneration or extrusion. Follicles with similar size and appearance were placed into 96‐well plates, with each well filled with 100 µL of α‐MEM containing 3% BSA, 10 mIU/ml of recombinant follicle‐stimulating hormone, along with 5 µg/mL insulin‐transferrin‐selenium (ITS‐G, 41400045, Gibco, USA), and 0.5% penicillin–streptomycin. Since the oocyte maturation process was found to be disturbed, we did not conduct further follicle maturation experiments. A half volume of the medium was changed every other day. Three separate experiments with a total of 80 follicles were included and divided into groups of 10 follicles. The appropriate RGDfK doses were determined by preliminary dose‐screening experiments (n = 3 biological replicates per dose group). Since secondary follicles are more sensitive to environmental stimuli than intact ovaries, we tested RGDfK doses of 0.5, 5, 50, 100, and 500 µM. Doses ≤0.5 µM had no effect on follicle survival; 5 and 50 µM reduced follicle growth rate after 7 days; 100 µM caused massive follicle death; 500 µM resulted in complete follicle degeneration. To capture dose‐dependent effects, we selected 5 µM (low), 50 µM (moderate), and 500 µM (high) for secondary follicle culture.

### 5.28 Isolation of Total RNA of Denuded Oocytes

Extraction of denuded oocytes’ RNA was achieved with the use of NucleoZOL according to the manufacture's manual. Briefly, 40 oocytes were mixed with 300 µL NucleoZOL and 120 µL RNase‐free water. Shake the tube vigorously for 15 s, and incubate on ice for 15 min. After centrifugation, Add a mixture of sodium acetate and Dr. GenTLE precipitation carriers to the supernatant for precipitation of the RNA. The following steps were similar to traditional RNA extraction process.

### 5.29 Isolation and Culture of Granulosa Cells

The ovarian tissues were placed in Leibovitz's L‐15 medium, and a 30‐gauge needle was employed to puncture the follicles. After centrifugation, granulosa cells were suspended and planted onto the six‐well plastic culture plates for primary culture system. Granulosa cells were supplemented with MaCoy 5A medium, which includes 3.7 mg/mL NaHCO3, 10% FBS, 100 IU/mL penicillin, and 100 mg/mL streptomycin, and then incubated at 37°C with 5% CO2.

### 5.30 Terminal Deoxynucleotidyl Transferase‐Mediated Deoxyuridine Triphosphate (TUNEL) Assay

According to the manufacturer's guidelines (C1089, Beyotime, China), the largest ovarian section was employed for apoptosis analysis. Follicles embedded in paraffin were cut into routine sections of 5 µm thickness. Each section was incubated in TUNEL reaction medium for one hour at 37°C in the dark. Once the reaction was halted, the sections were washed and stained with DAPI for 5 min. Using a fluorescence microscope (Olympus, Japan), TUNEL‐positive cells in ovarian tissues from each group were observed and analyzed.

### 5.31 Cell Viability Assay

GCs were seeded in 96‐well plates and grown to 90% confluency over 4 days. After the treatments, 10 µL of CCK‐8 assay reagent (CCK‐8; Dojindo Laboratories, CK04) was added to each well with 100 µL of medium, and incubated in the dark at 37°C for 2 h. The optical density at 450 nm was measured using a microplate spectrophotometer (Thermo Fisher Scientific, Camarillo, CA, USA) to evaluate the formation of formazan.

### 5.32 Flow Cytometry Assay

The apoptosis of GCs was assessed through flow cytometry with a FITC Annexin V/PI Apoptosis Detection Kit. In short, cells were plated in 6‐well plates at a density of 4 × 10^5^. After a 48‐hour incubation, the cells were washed two to three times with cold PBS and stained with FITC Annexin V and propidium iodide for 15 min at room temperature.

### 5.33 Culture of Immortalized Human GCs

KGN cells were grown in DMEM High Glucose (HyClone, Logan City, UT) with 10% fetal bovine serum (BI, Beit‐Haemek, Israel), 100 U/mL penicillin G, and 0.1 mg/mL streptomycin sulfate (SV30010; HyClone, Logan City, UT). COV434 cells were maintained in DMEM/F12 medium (HyClone) that included 10% fetal bovine serum (HyClone) and 100 U/mL penicillin G.

### 5.34 Weighted Gene Co‐Expression Network Analysis (WGCNA)

This study was carried out using the gene profiles generated by RNA‐seq experiment (ArrayExpress NO: E‐MTAB‐1587) [57, 58]. Oocyte datasets were independently developed using this method. After transforming the Pearson correlation matrix into a connection strength matrix using a power of 10, the topological overlap was determined to evaluate the network's interconnected score. Genes were grouped using average linkage hierarchical clustering based on the dissimilarity measure of their network connection strengths, calculated as 1 minus the topological overlap. We identified 19 modules in the oocyte datasets and the merging threshold was set at 0.25. The WGCNA R package, a freely accessible statistical analysis tool, along with R tutorials for building a weighted gene co‐expression network, have been detailed earlier. We focused on differentially expressed genes rather than all annotated genes to more accurately depict molecular events during follicular development.

### 5.35 Ovarian Biopsies

The study incorporated ovarian tissue samples from patients across a range of ages. All adult participants were undergoing laparoscopic surgery for benign gynecologic diseases that did not impact the ovaries. Use of human ovarian tissues were approved by the Institutional Review Board of Huazhong University of Science and Technology (ID: TJ‐IRB20210319). Written informed consents were given by all patients or their parents. Ovarian tissue samples were obtained from 14 patients. Five of these patients were fetuses (aged 22–34 weeks) due to abortion, while the remaining nine were adults (aged 22–36 years) with malignant conditions.

### 5.36 Collagen Image Analysis

Using a polarized microscope (NIKON Eclipse CI), sections stained with Sirius red were viewed. Images were captured under polarized and brightfield lighting to acquire morphological and developmental insights, with consistent magnification. To investigate fiber organization around follicles, their borders were delineated manually and selected as area of interest. CT‐FIRE V2.0 Beta was used to ensure the reproducible and objective results of histological slide [18, 59].

### 5.37 Statistical Analysis

All experiments were performed in triplicates under independent and controlled conditions. Data were presented as mean ± standard deviation (SD). Unpaired Student's t test (2‐sided) was used for statistical analysis between two groups. Pearson's chi‐squared test was applied for statistical analysis of percentage data. One‐way or two‐way ANOVA was used for multiple group comparisons. Pearson correlation analysis was used to calculate the correlation coefficient. P < 0.05 was considered statistically significant. GraphPad Prism 8.0 (GraphPad Software) was used for statistical analysis.

## Author Contributions

T.W., M.W., S.X.W., and J.J.Z. designed the research and wrote the manuscript. T.W., Y.Y.G., Q.Q.Z., K.B.N., Y.C., D.X.H., Y.Z.F., and X.Y. performed the experiments. T.W., T. J., K.B.N., and X.N.T. analyzed and interpreted the data. S.X.W., T.W. got the funding. S.X.W. supervised the entire study. All authors reviewed the manuscript.

## Funding

This work is supported by the grants from the National Key Research and Development Program of China (NO.2022YFC2704100), and National Natural Science Foundation of China (NO. 82301849, 82371648).

## Conflicts of Interest

The authors declare no conflicts of interest.

## Supporting information




**Supporting File 1**: advs74771‐sup‐0001‐SuppMat.docx.

**Supporting File 2**: advs74771‐sup‐0002‐Tables S1‐S3.xlsx.

**Supporting File 3**: advs74771‐sup‐0003‐Data.zip.

## Data Availability

The data that support the findings of this study are available from the corresponding author upon reasonable request.;
