# Supplementary material for: Ovarian Matrisome Dynamics and αvβ3‐Mediated Regulation in Early Follicular Development
Source: Adv Sci (Weinh). 2026 Mar 14;13(29):e07314. doi: 10.1002/advs.202507314 (PMC13205675; doi:10.1002/advs.202507314)
Supplement: Supplementary file 1 — Supporting File 1: advs74771‐sup‐0001‐SuppMat.docx. [file ADVS-13-e07314-s002.docx]

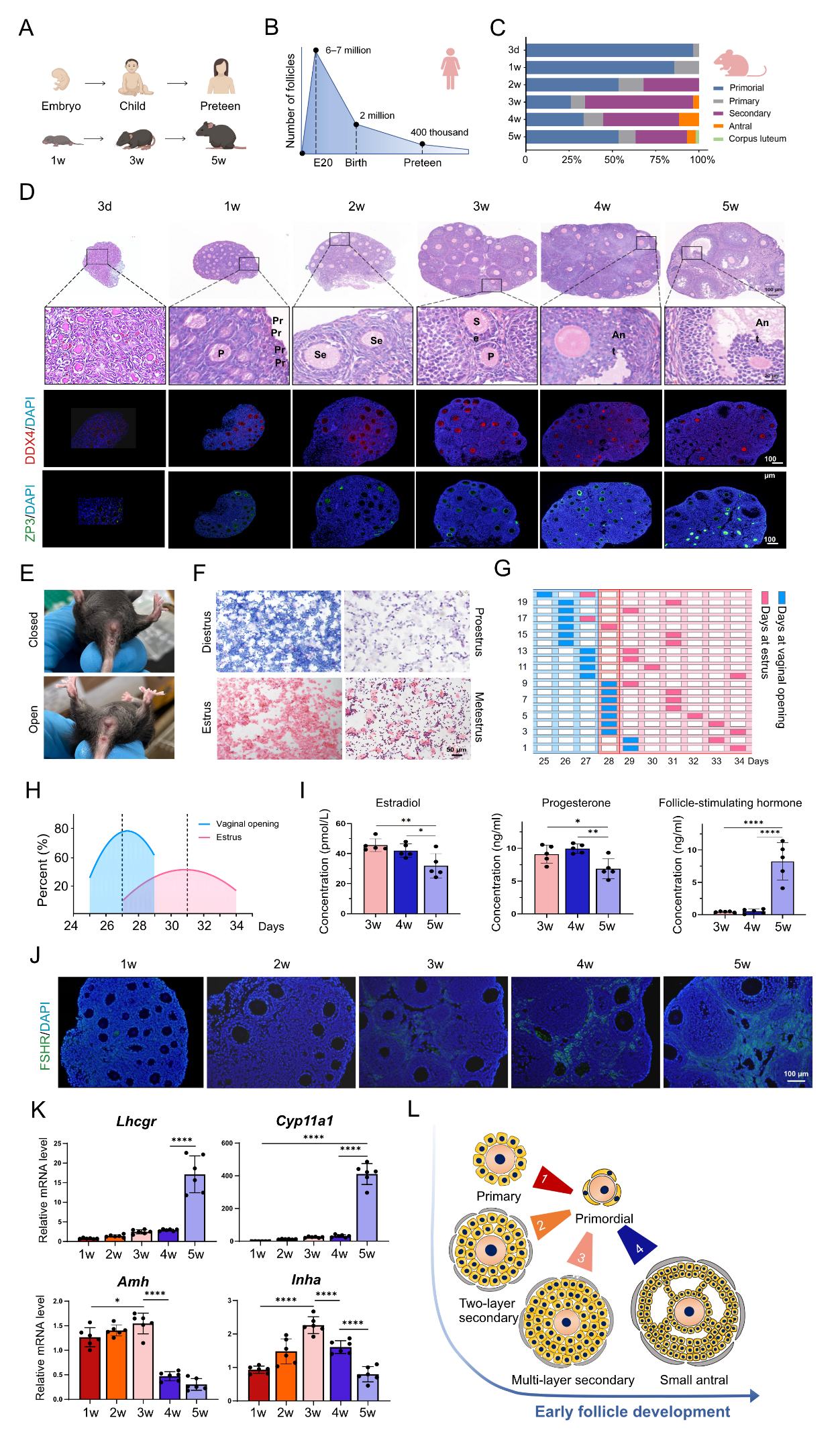


## Figure S1. Prepubertal development of ovaries in C57BL/6J mice.

(A) Schematic of age correspondence between humans and mice in terms of ovarian function. (B) Numerical alteration of follicles in human ovaries. (C) Quantitative analysis of follicle stage ratios in prepubertal mouse ovaries (3 days old to 5 weeks old, n=5 per age group). (D) Representative images of follicles by hematoxylin and eosin (H&E) staining and immunofluorescence staining for DDX4 (oocyte marker, green) and ZP3 (growing follicle marker, red) of mouse ovaries at different ages. Nuclei were counterstained with DAPI (blue). (E) Representative images of vaginal opening and estrus smears at different postnatal days. (F) Classification of estrus stages based on vaginal smear cellular composition. (G) Timing of vaginal opening and first estrus in individual mice (n=20). (H) Non-linear regression analysis of vaginal opening rate (blue line) and estrus occurrence rate (red line) from postnatal day 24 to 34. (I) Concentrations of serum estradiol, progesterone, and follicle stimulating hormone in 3–5-week-old mice (FSH, n=5 each hormone). (J) Distribution of FSH receptor (FSHR) in 1–5-week-old mouse ovaries. (K) Relative mRNA levels of steroidogenesis and ovarian development related markers (n=5). (L) Schematic illustrating the timeline of early follicular development (EFD) in prepubertal mice.

Data are presented as mean ± standard deviation (SD). Statistical analysis was performed using one-way ANOVA (I, K). All tests were two-tailed. *, P < 0.05; **, P < 0.01; ****, P < 0.0001.

Abbreviations: Ant, antral follicles; Pr, primordial follicles; P, primary follicles; Se, secondary follicles; ns, not significant.


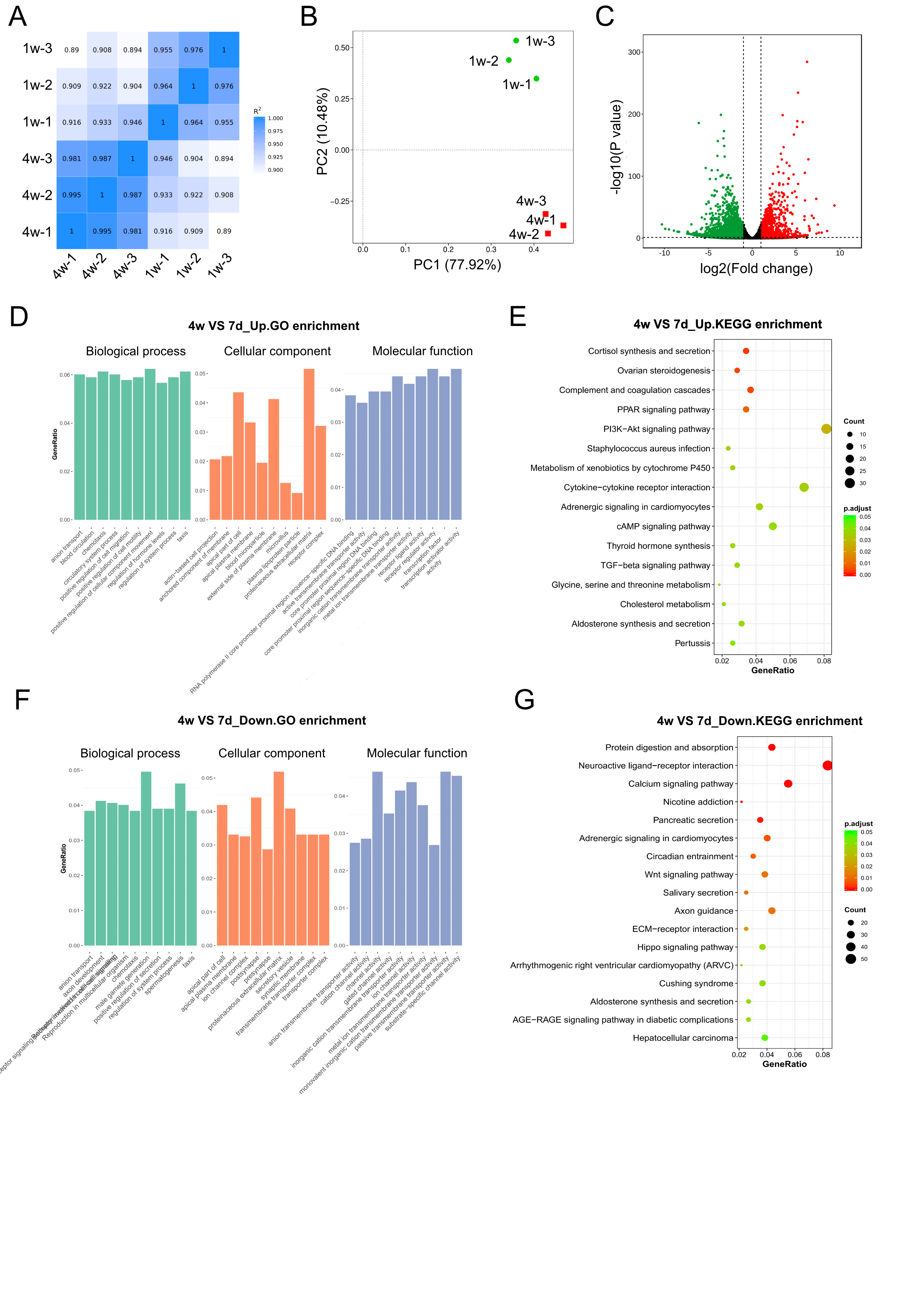


## Figure S2. Transcriptomic profiling reveals dynamic changes in gene expression during ovarian development from 1 week to 4 weeks of age.

(A) Correlation matrix heatmap of transcriptomic profiles across samples. The color scale represents Pearson correlation coefficients. (B) Principal component analysis plot of transcriptomic data. (C) Volcano plot showing differentially expressed genes (DEGs) between 4-week-old and 1-week-old ovaries. (D) Gene Ontology enrichment analysis of upregulated DEGs in 4-week-old vs. 1-week-old ovaries. (E) Kyoto Encyclopedia of Genes and Genomes (KEGG) pathway enrichment analysis of upregulated DEGs. (F) GO enrichment analysis of downregulated DEGs in 4-week-old vs. 1-week-old ovaries. (G) KEGG pathway enrichment analysis of downregulated DEGs. Data were generated from RNA sequencing of ovarian tissues (n = 3 biological replicates per group). Statistical significance for DEGs was determined using Limma R package with adjusted P < 0.05 and |log2(foldchange| > 1.

##
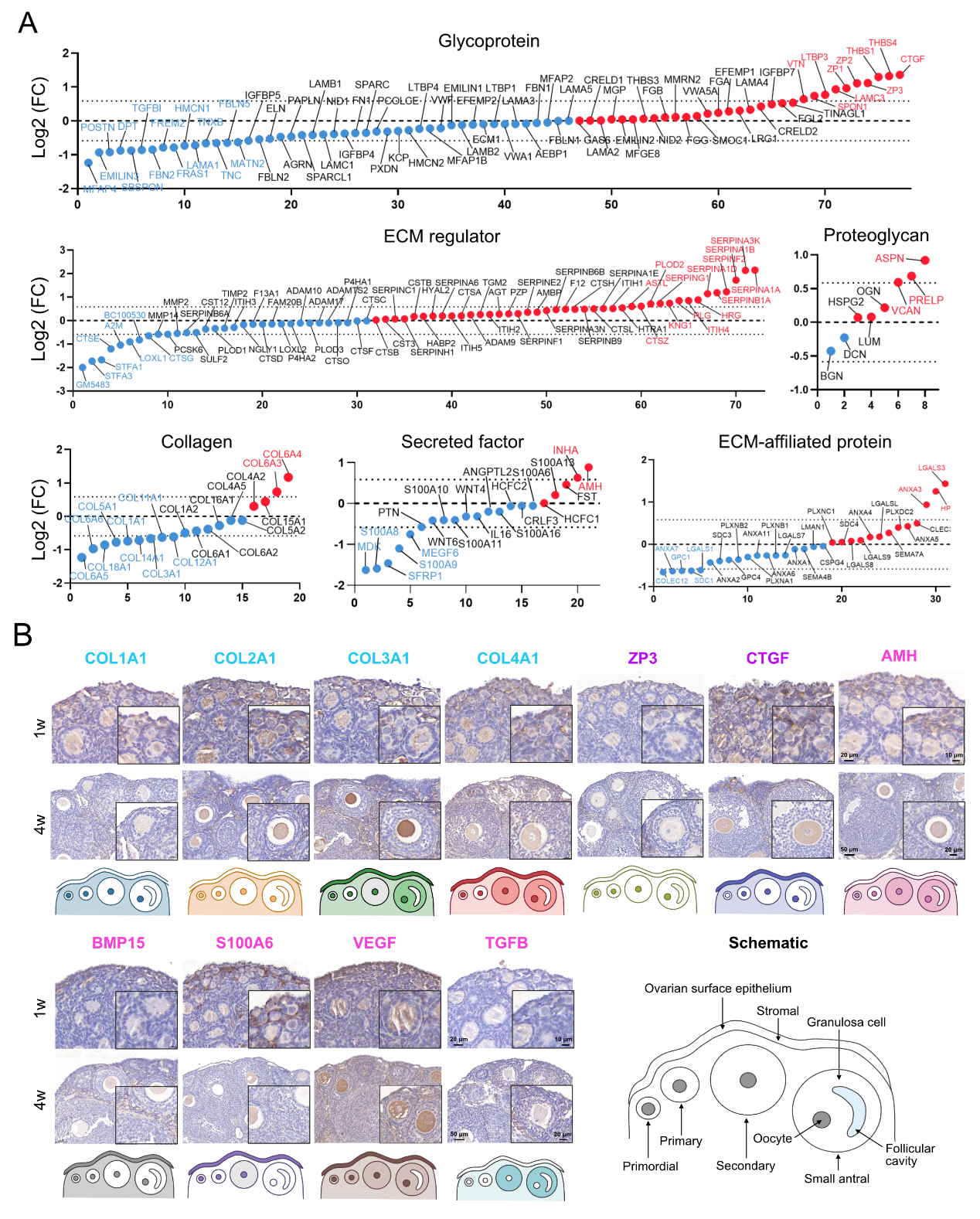
Figure S3. Classification and distribution of matrisome proteins in ovaries.

(A) Proteomic levels of differentially expressed matrisome proteins in 1-week-old vs. 4-week-old mouse ovaries. (B) Distributions of differential expressed matrisome proteins during EFD, with insets providing higher - magnification views of follicular regions. Schematic on the right illustrates ovarian components (ovarian surface epithelium, stroma, granulosa cells, oocyte, follicular cavity) and follicle stages (primordial, primary, secondary, small antral).


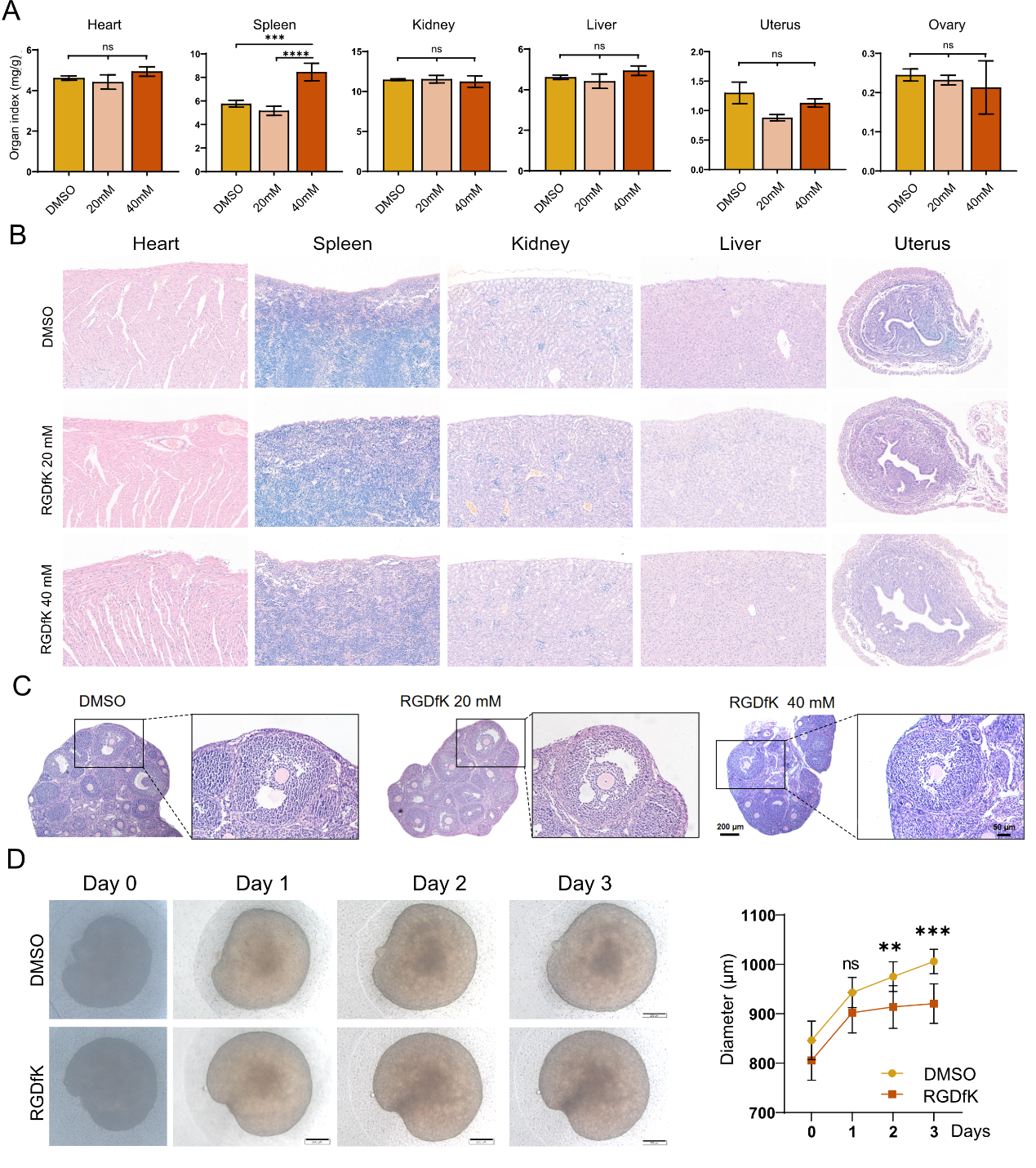


## Figure S4. General evaluation of mice and growth of neonatal ovaries.

(A-B) Organ indexes and representative H&E staining of heart, spleen, kidney, liver, uterus and ovary (n=6 per group). (C) Representative H&E staining of ovaries for puberty score assessment. (D) Volume change of neonatal ovaries during in vitro culture. Ovarian volume was calculated based on length and width measurements using ImageJ. Data are presented as mean ± SD. Statistical analysis was performed using one-way ANOVA (A) or two-way ANOVA (D). All tests were two-tailed. **, P < 0.01; ***, P < 0.001; ns, not significant.


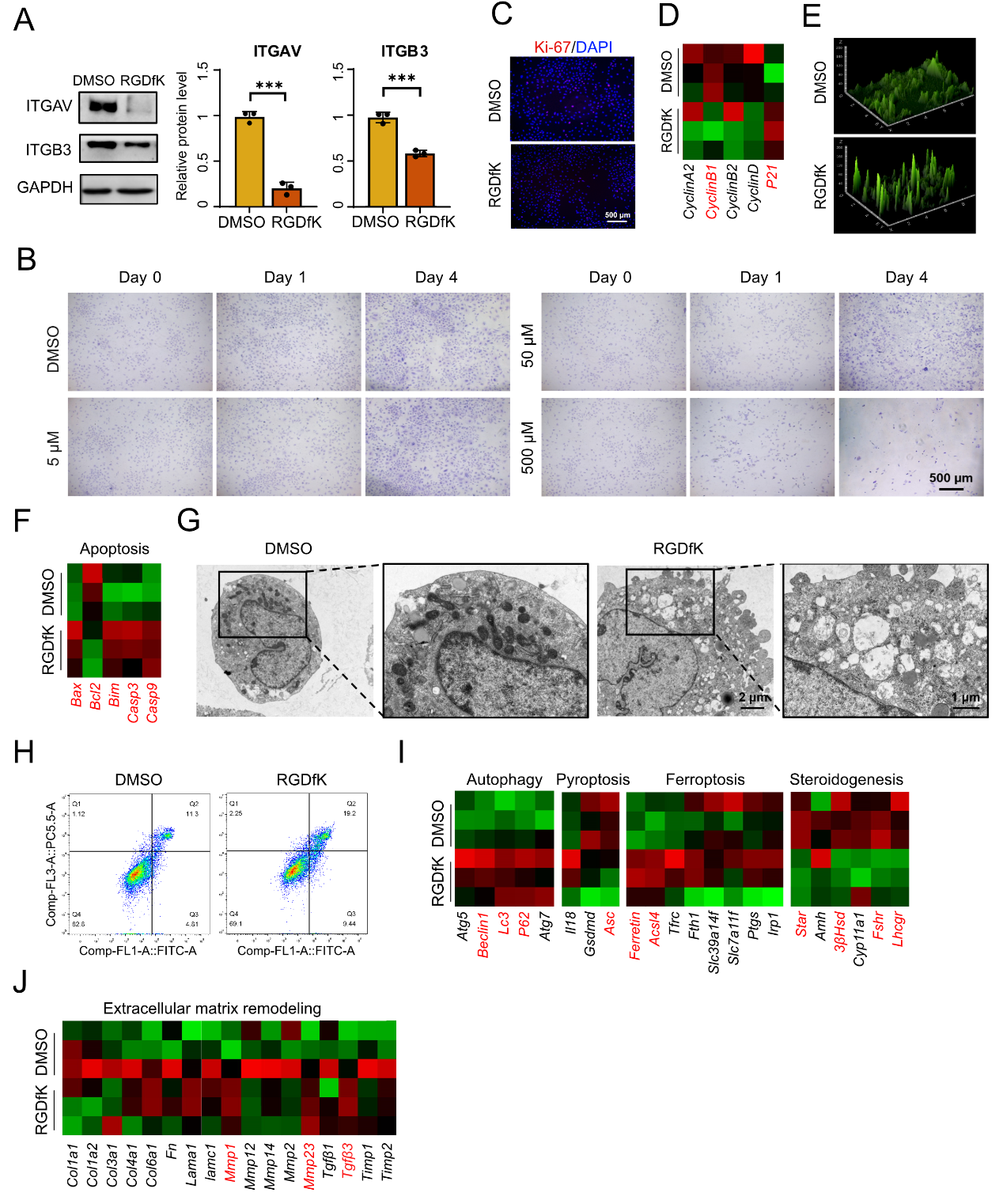


## Figure S5. Effects of integrin inhibition on GCs.

(A) Western blotting validation of ITGAV and ITGB3 protein downregulation in RGDfK-treated primary GCs. (B) Representative images of crystal violet-stained KGN cells. (C) Ki-67 staining for primary GC proliferation. (D) Heatmap of cell cycle-related gene expression. (E) 3D reconstruction of cytoskeleton showing microfilament depolymerization and microtubule breakage in RGDfK-treated GCs. (F) mRNA levels of apoptosis in GCs. (G) TEM images of primary GCs, showing apoptotic bodies in RGDfK-treated groups. (H) Flow cytometry plots showing apoptosis analysis in DMSO- and RGDfK-treated cells. (I-J) mRNA levels of autophagy, pyroptosis, ferroptosis, and ECM remodeling encoding genes.
