# Supplementary material for: Ovarian Matrisome Dynamics and αvβ3‐Mediated Regulation in Early Follicular Development
Source: Adv Sci (Weinh). 2026 Mar 14;13(29):e07314. doi: 10.1002/advs.202507314 (PMC13205675; doi:10.1002/advs.202507314)

Full set of unmodified, raw data

CTGF Figure 1I


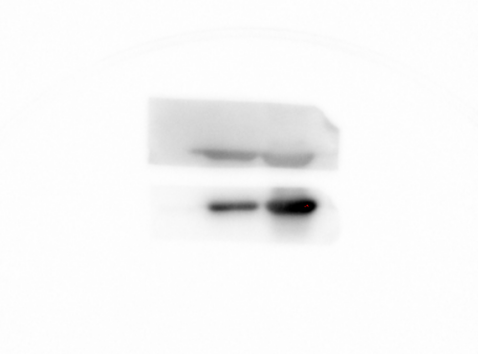

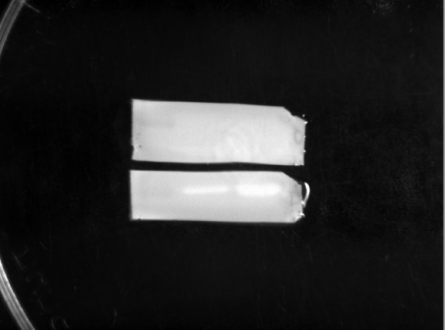


LAMA1 Figure 1I


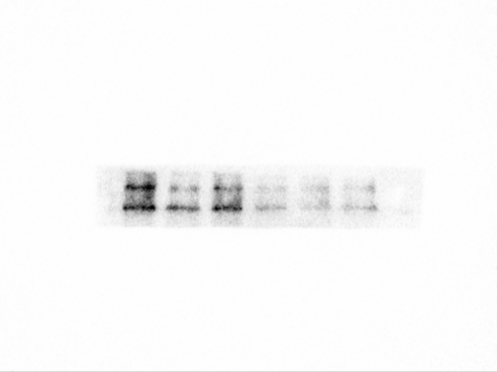


COL1 Figure 1I


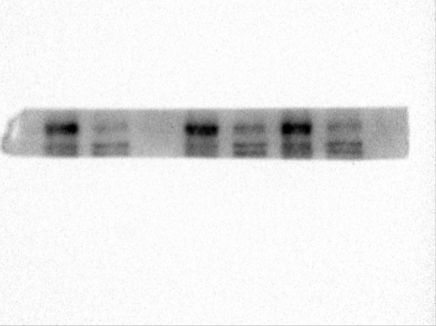


COL3 Figure 1I


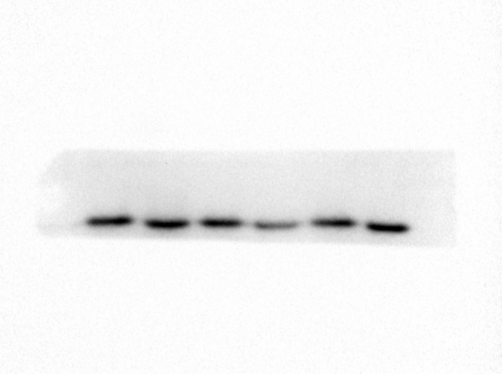


ZP3 Figure 1I


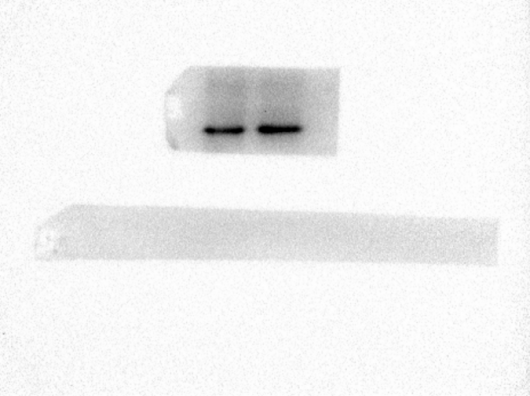


ITGAV Figure 5G


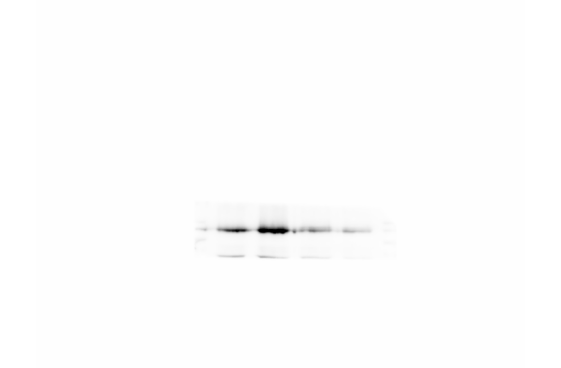

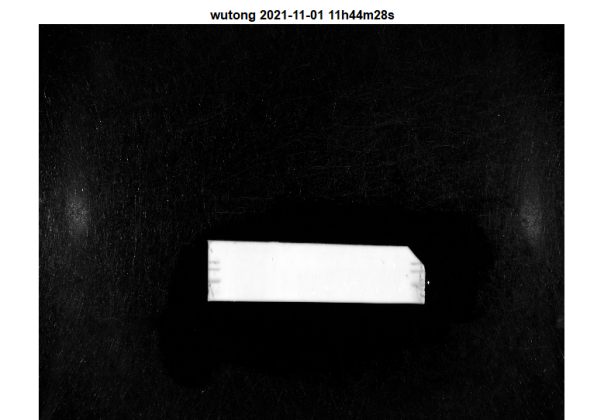


ITGB3 Figure 5G


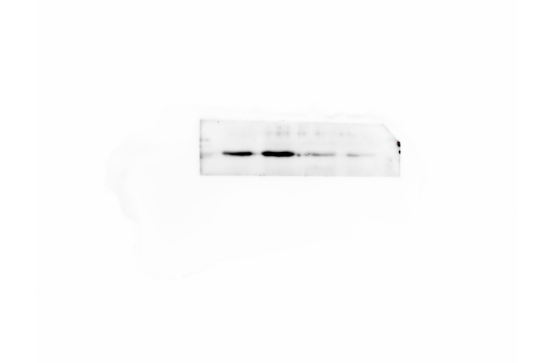

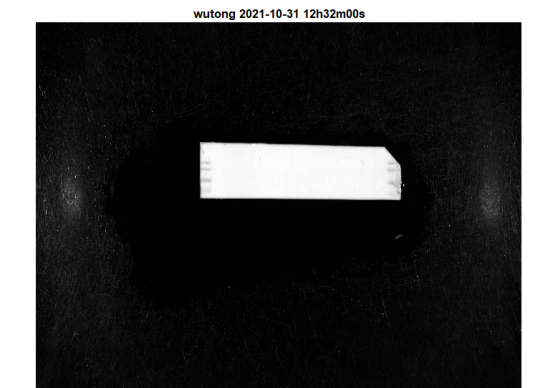


**ACTB Figure 5G**


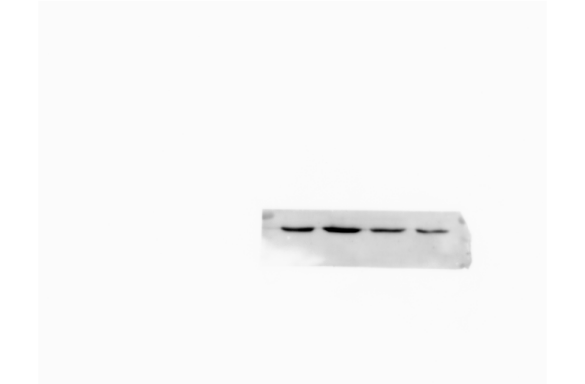

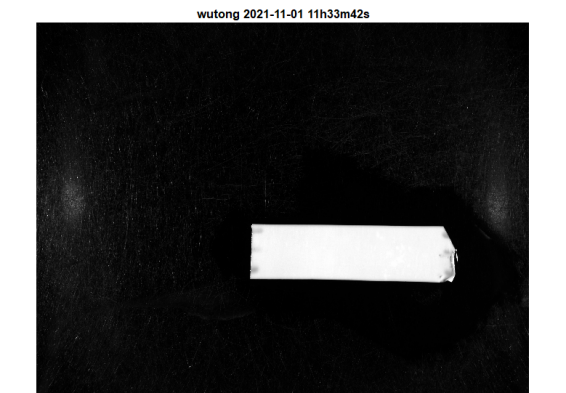


GAPDH Figure 5G


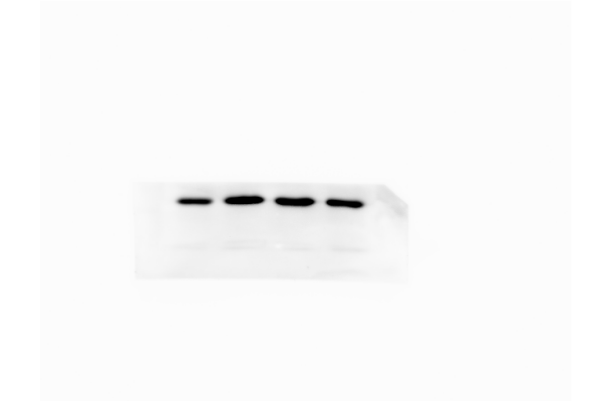

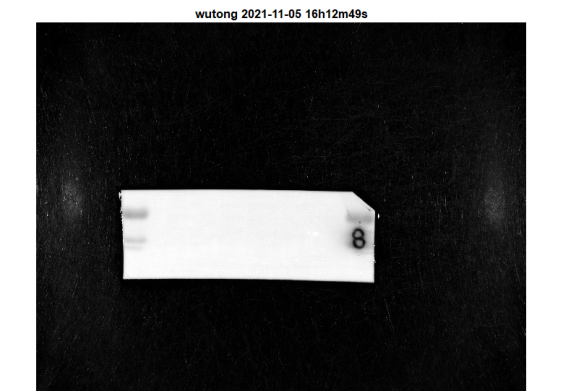


YAP Figure 5K


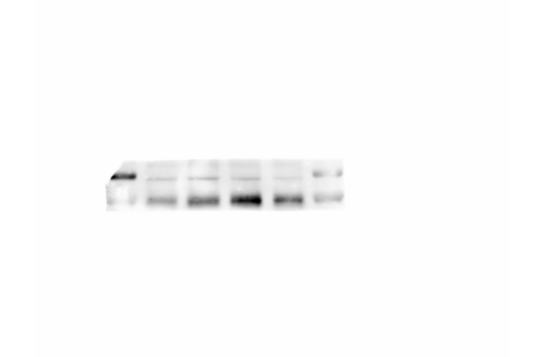

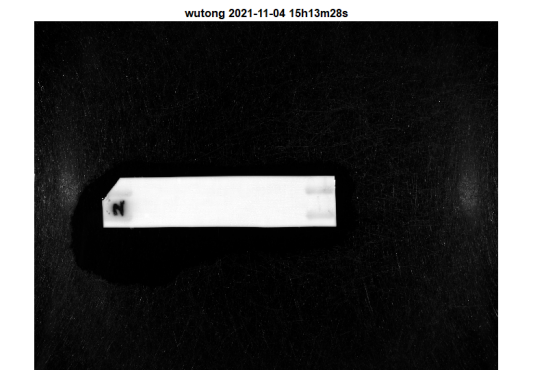


MST1 Figure 5K


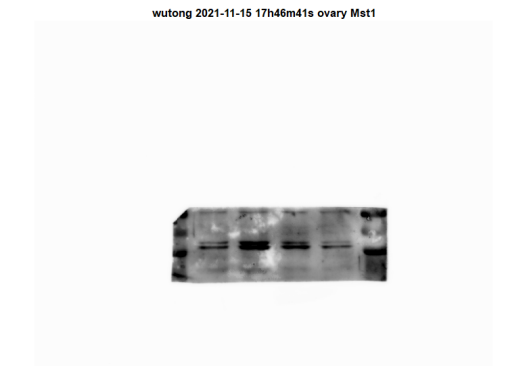

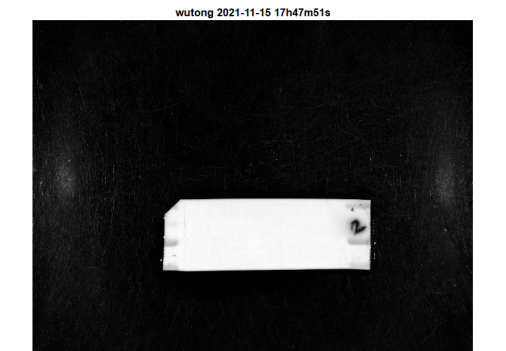


PUMA Figure 5K


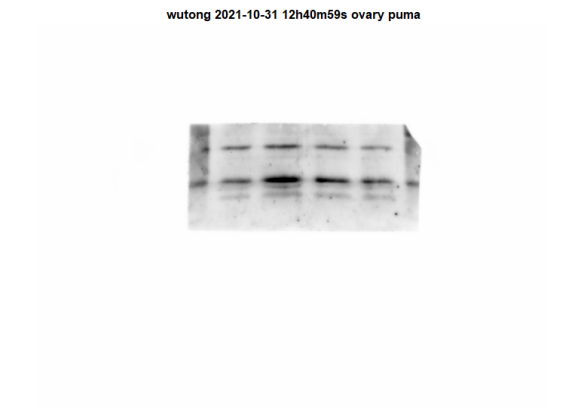

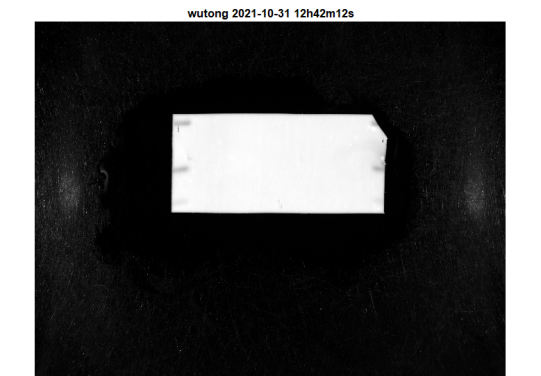


GAPDH Figure 5K


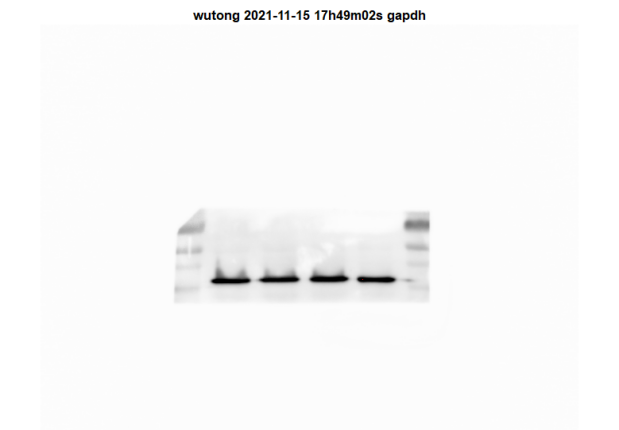

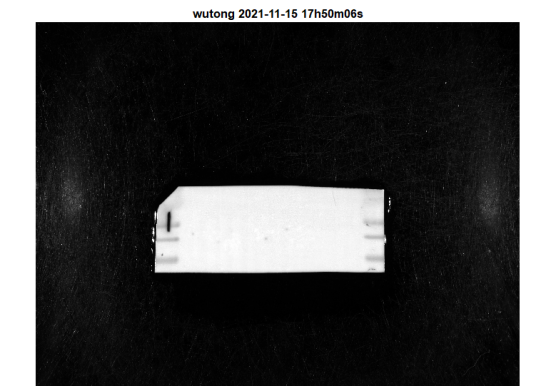


mTOR Figure 5M


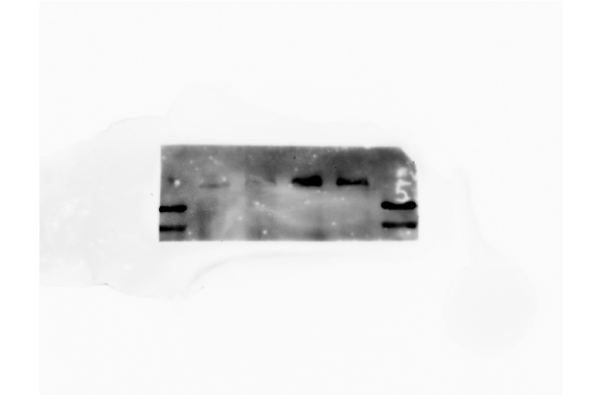

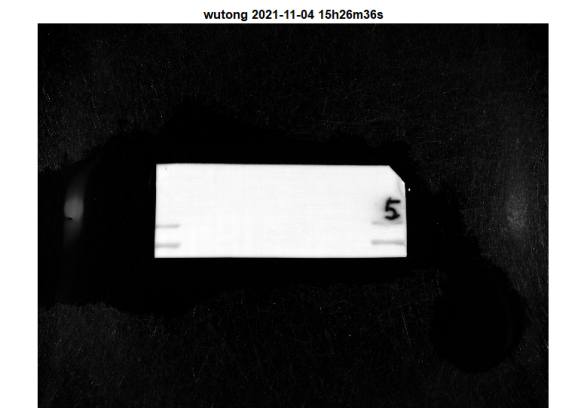


p-mTOR Figure 5M


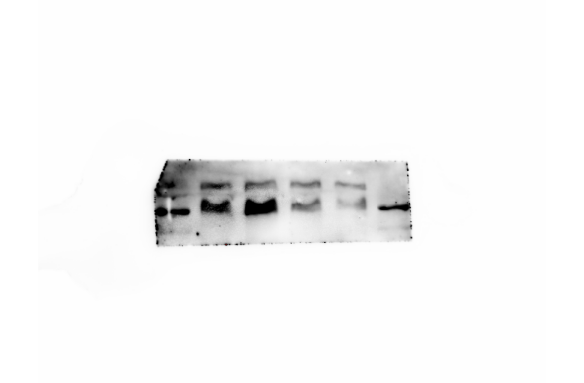

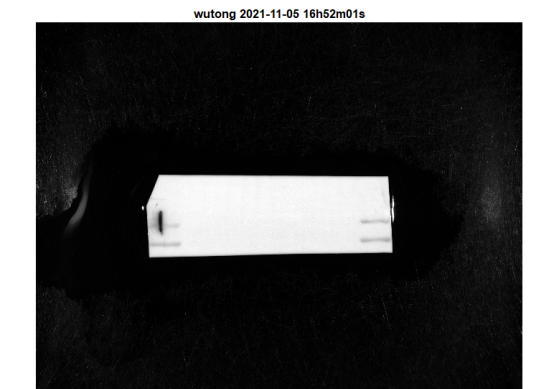


PTEN Figure 5M


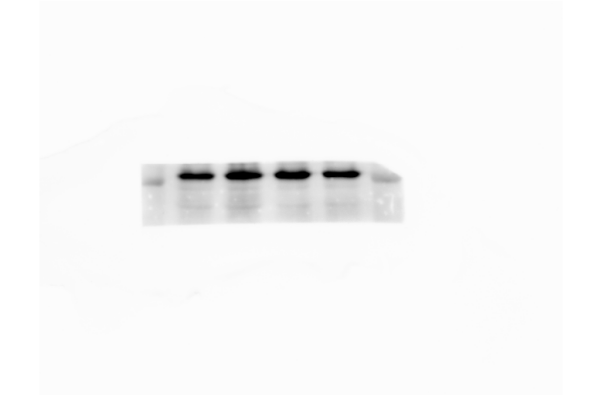

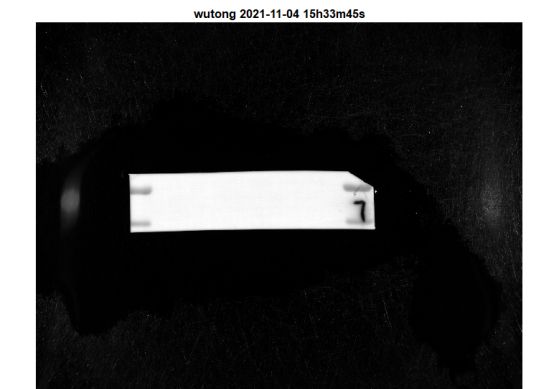


FOXO3a Figure 5M


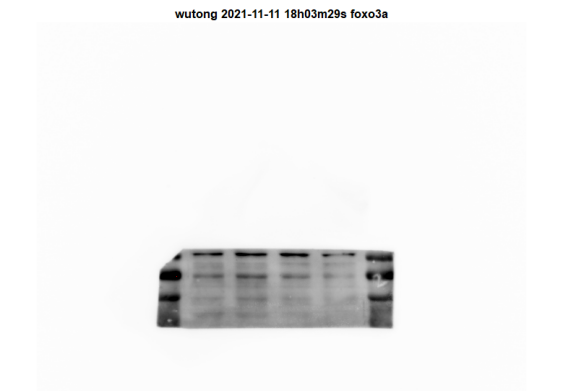

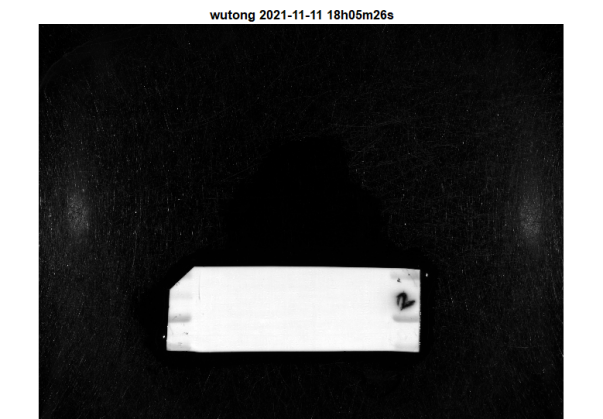


GAPDH Figure 5M


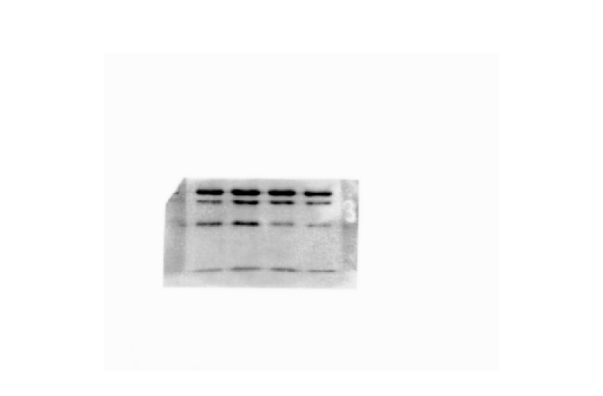

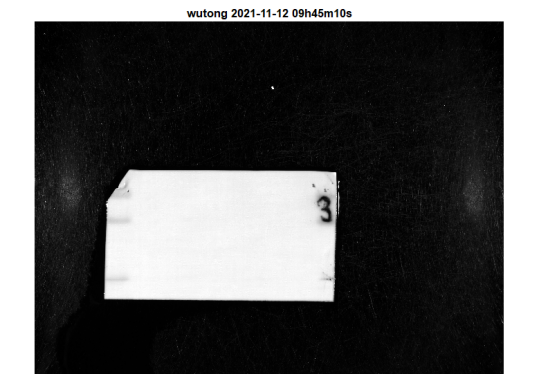


BCL2 Figure 5N


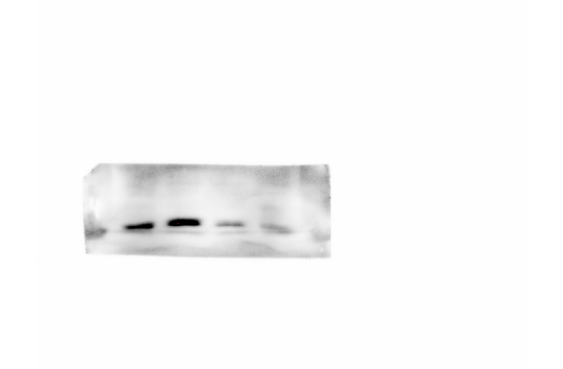

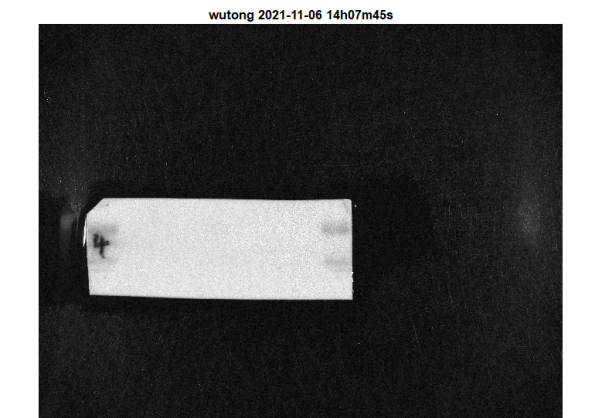


CASP3 Figure 5N


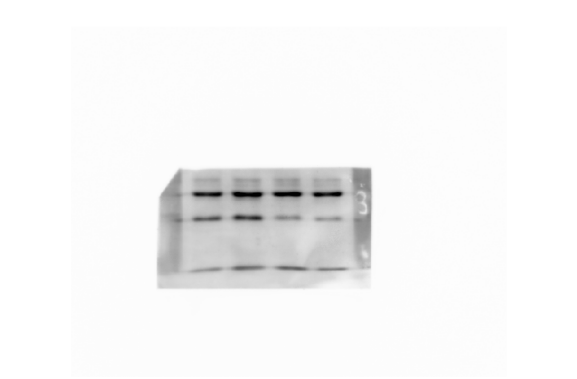

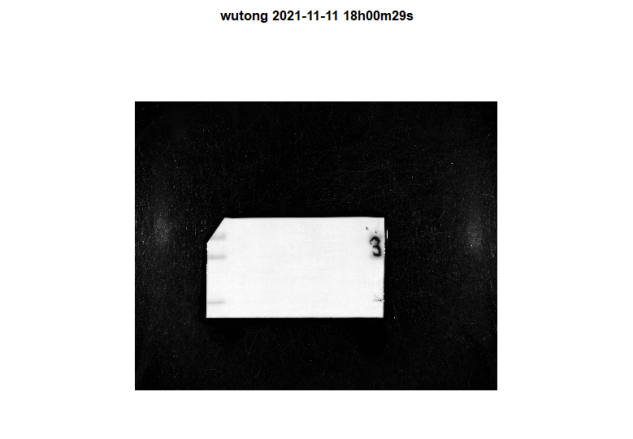


GAPDH Figure 5N


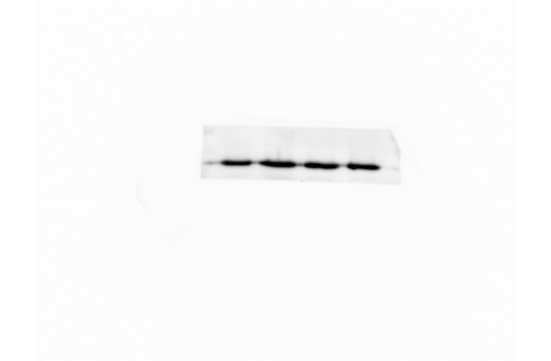

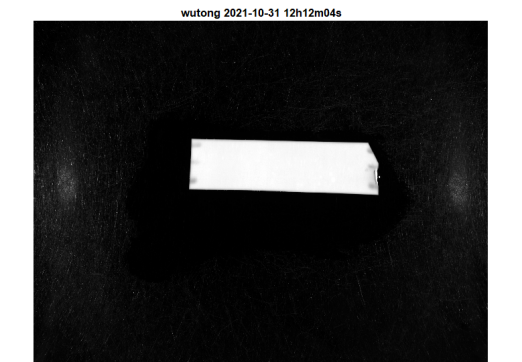


RHOA Figure 6M


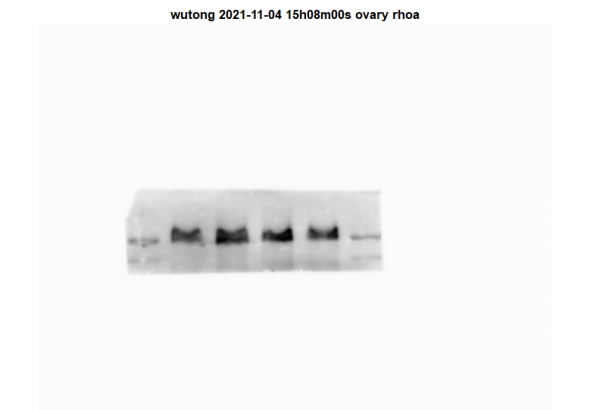

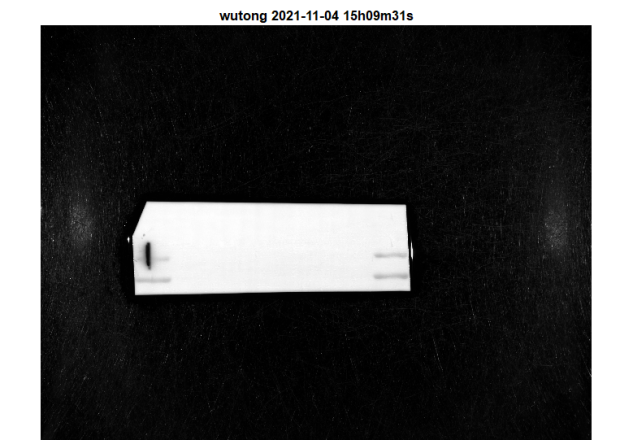


ROCK Figure 6M


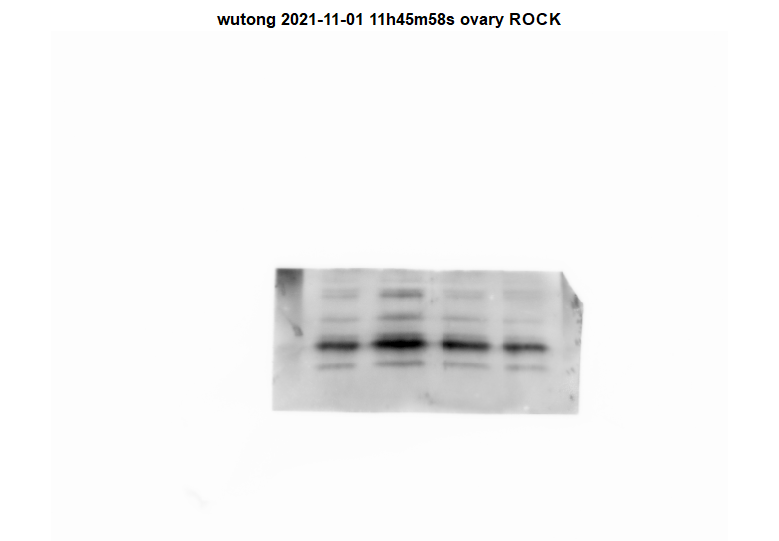

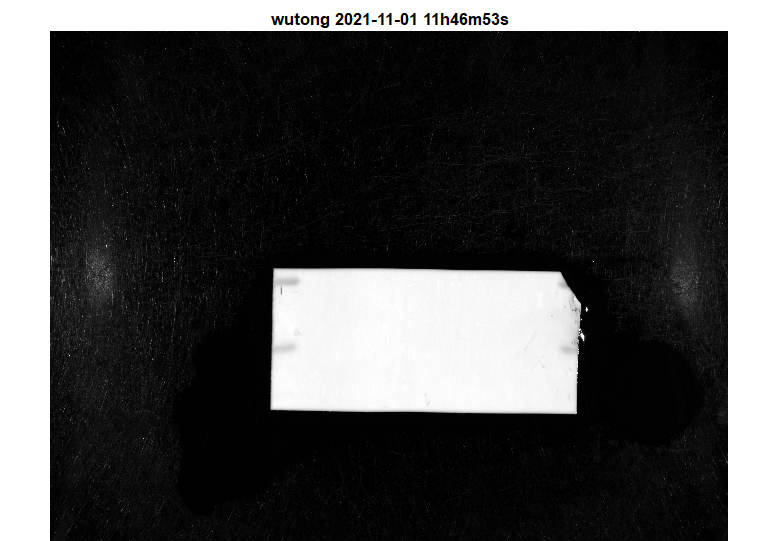

Supplement: Supplementary file 3 — Supporting File 3: advs74771‐sup‐0003‐Data.zip. [file ADVS-13-e07314-s001.zip › 2026.2.22 Raw data.docx]
