# Supplementary figures and images for: Ovarian Matrisome Dynamics and αvβ3‐Mediated Regulation in Early Follicular Development
Source: Adv Sci (Weinh). 2026 Mar 14;13(29):e07314. doi: 10.1002/advs.202507314 (PMC13205675; doi:10.1002/advs.202507314)

Western blotting for 7d and 4w ovaries.


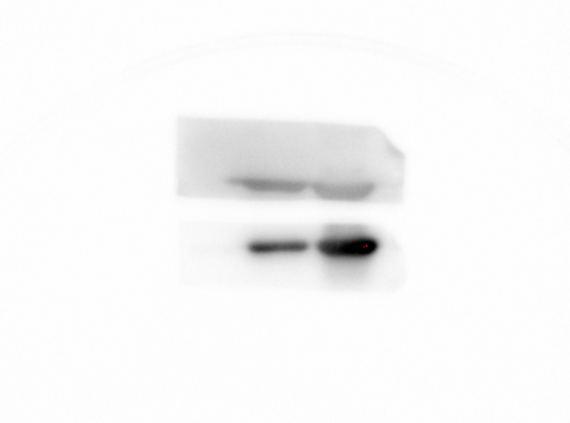

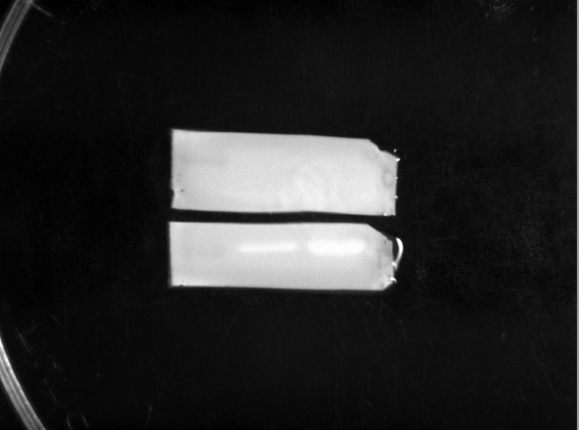

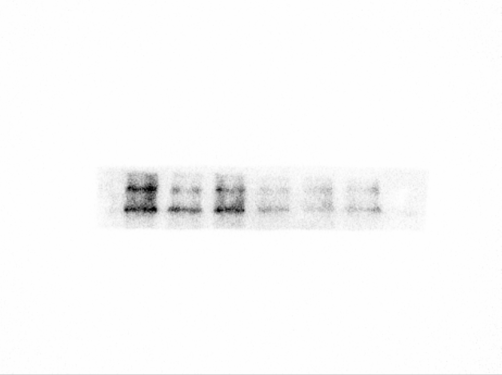

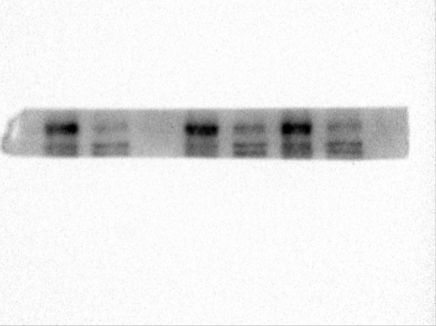


CTGF

LAMA1

COL1A1


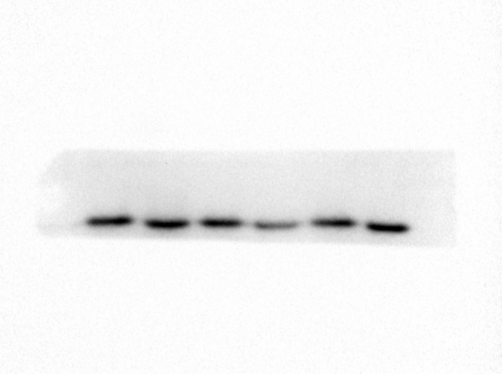


COL3A1


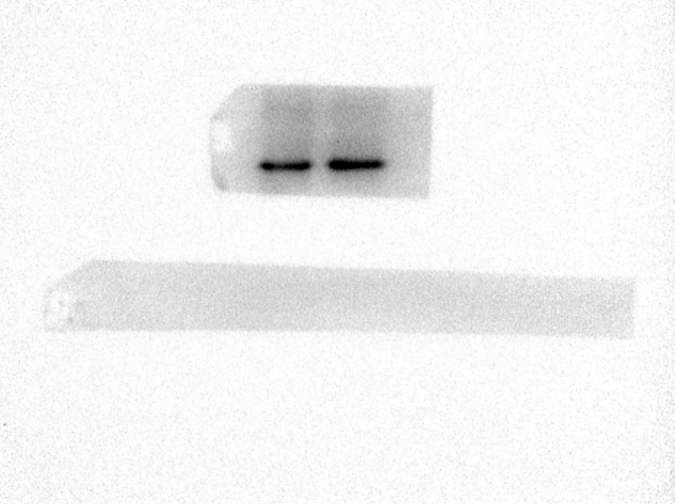


ZP3

Supplement: Supplementary file 3 — Supporting File 3: advs74771‐sup‐0003‐Data.zip. [file ADVS-13-e07314-s001.zip › Supplementary figure.docx]
